# Construction of a chiral artificial enzyme used for enantioselective catalysis in live cells†

**DOI:** 10.1039/d0sc03082a

**Published:** 2020-09-23

**Authors:** Ya Zhou, Weili Wei, Fengchao Cui, Zhengqing Yan, Yuhuan Sun, Jinsong Ren, Xiaogang Qu

**Affiliations:** Laboratory of Chemical Biology and State Key Laboratory of Rare Earth Resource Utilization, Changchun Institute of Applied Chemistry, Chinese Academy of Sciences Changchun Jilin 130022 China xqu@ciac.ac.cn; University of Science and Technology of China Hefei Anhui 230026 China

## Abstract

Nanozymes as a newcomer in the artificial enzyme family have shown several advantages over natural enzymes such as their high stability in harsh environments, facile production on large scale, long storage time, low costs, and higher resistance to biodegradation. However, compared with natural enzymes, it is still a great challenge to design a nanozyme with high selectivity, especially high enantioselectivity. It is highly desirable and demanding to develop chiral nanozymes with high and on-demand enantioselectivity for practical applications. Herein, we present an unprecedented approach to construct chiral artificial peroxidase with ultrahigh enantioselectivity. Inspired by the structure of the natural enzyme horseradish peroxidase (HRP), we have constructed a series of stereoselective nanozymes (Fe_3_O_4_@Poly(AA)) by using the ferromagnetic nanoparticle (Fe_3_O_4_ NP) yolk as the catalytic core and amino acid-appended chiral polymer shell as the chiral selector. Among them, Fe_3_O_4_@Poly(d-Trp) exhibits the highest enantioselectivity. More intriguingly, their enantioselectivity will be readily reversed by replacing d-Trp with l-Trp. The selectivity factor is up to 5.38, even higher than that of HRP. Kinetic parameters, dialysis experiments, and molecular simulations together with activation energy reveal that the selectivity originates from the d-/l-Trp appended polymer shell, which can result in better affinity and catalytic activity to d-/l-tyrosinol. The artificial peroxidases have been used for asymmetric catalysis to prepare enantiopure d- or l-enantiomers. Besides, by using fluorescent labelled FITC-tyrosinol_L_ and RhB-tyrosinol_D_, the artificial peroxidases can catalyze green or red fluorescent chiral tyrosinol to selectively label live yeast cells among yeast, *S. aureus*, *E. coli* and *B. subtilis* bacterial cells. This work opens a new avenue for better design of stereoselective artificial enzymes.

## Abstract 2

A yolk–shell stereoselective nanozyme is designed with a chiral selector. Nanozyme with D-/L-tryptophan possesses high selectivity towards D-/L-tyrosinol and can catalyze chiral tyrosinol to selectively label live yeast cells.

## Graphical abstract

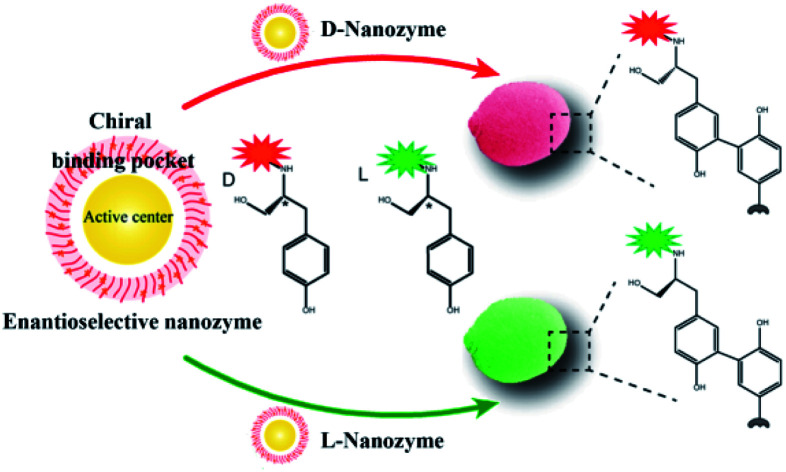

## Introduction

The design and synthesis of artificial enzymes or enzyme mimetics with high activity and selectivity have been the focus in the pursuit of alternatives for natural enzymes since the middle of the last century.^1^ In the past 10 years, as a new generation of artificial enzymes, nanomaterials with enzyme–mimetic activity, named nanozymes, have attracted great interest because of their many advantages over natural enzymes, including high stability in high temperature and harsh environments, facile production on a large scale, long storage time, low costs, and higher resistance to biodegradation.^2^ Among them, the Fe_3_O_4_ nanozyme is a classical nanomaterial with intrinsic peroxidase-like activity and was first reported by Yan and coworkers in 2007.^3^ Different types of nanozymes have been developed and successfully used for biosensing,^3*a*,4^ cancer therapy,^5^ combating bacteria and biofilms,^6^ detoxification,^7^ and cytoprotection.^8^ Although promising, compared with natural enzymes, rational design and synthesis of nanozymes are still challenging,^9,10^ especially for the design of a chiral nanozyme with high selectivity.^11^ Therefore, it is important and necessary to develop chiral nanozymes with high and on-demand enantioselectivity for biosynthesis and practical applications.^12^

**Scheme 1:**
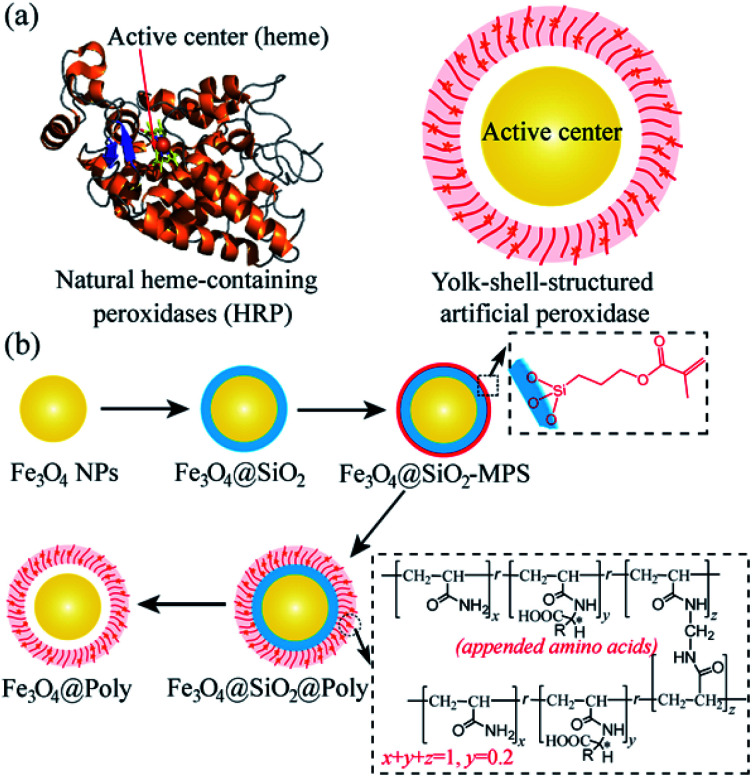
(a) Representative structures of natural heme-containing peroxidase (left) and yolk–shell-structured artificial peroxidase (right). (b) Preparation procedures of the yolk–shell-structured artificial peroxidase.

In natural enzymes, such as horseradish peroxidase (HRP, Scheme 1a, left), stereoselective reactions can be realized in the catalytic site (heme) with the assistance of proximally arrayed l-amino acid residues.^13^ The utilization of chiral amino acids as chiral selectors has also been proved to be effective for the construction of chiral nanozymes.^11*d*,*e*^ However, the previously reported chiral nanozymes relied on the direct modification of amino acids on the surface of nanoparticles. The density of the chiral selector is relatively low, resulting in low selectivity.^11*d*,*e*^ Besides, part of the active sites are covered by direct modification, which can hinder their activity.^11*d*,*e*^ Thus, a new modification strategy is desirable for the design of chiral nanozymes. The yolk–shell structure has been confirmed to be powerful for the construction of catalysts and this structure can avoid direct covalent bonding of amino acids blocking the central catalytic site of nanozymes.^14^ In addition, plenty of chiral amino acids can be introduced into the polymer shell to realize high efficiency chiral recognition.^15^ Inspired by the natural enzyme and advantages of the yolk–shell structure, a yolk–shell chiral nanozyme is designed by using Fe_3_O_4_ nanoparticle (NP) yolk as the catalytically active peroxidase-mimic and amino acid-appended polymer shell as the selector for chiral recognition (Fe_3_O_4_@Poly(AA), where Poly(AA) refers to polyacrylate(amino acid), Scheme 1a, right). The Poly(AA) shell allows selective access of chiral tyrosinol to the surface of peroxidase-like cores, thus yielding enantioselective artificial peroxidase. Scheme 1b illustrates well the synthesis process of Fe_3_O_4_@Poly(AA) *via* four steps, including preparing Fe_3_O_4_ NPs, coating Fe_3_O_4_ with a silica shell (Fe_3_O_4_@SiO_2_), modifying Fe_3_O_4_@SiO_2_ with a chiral polymer shell (Fe_3_O_4_@SiO_2_@Poly(AA)), and removing the silica shell to form yolk–shell Fe_3_O_4_@Poly(AA).

## Results and discussion

**Fig. 1:**
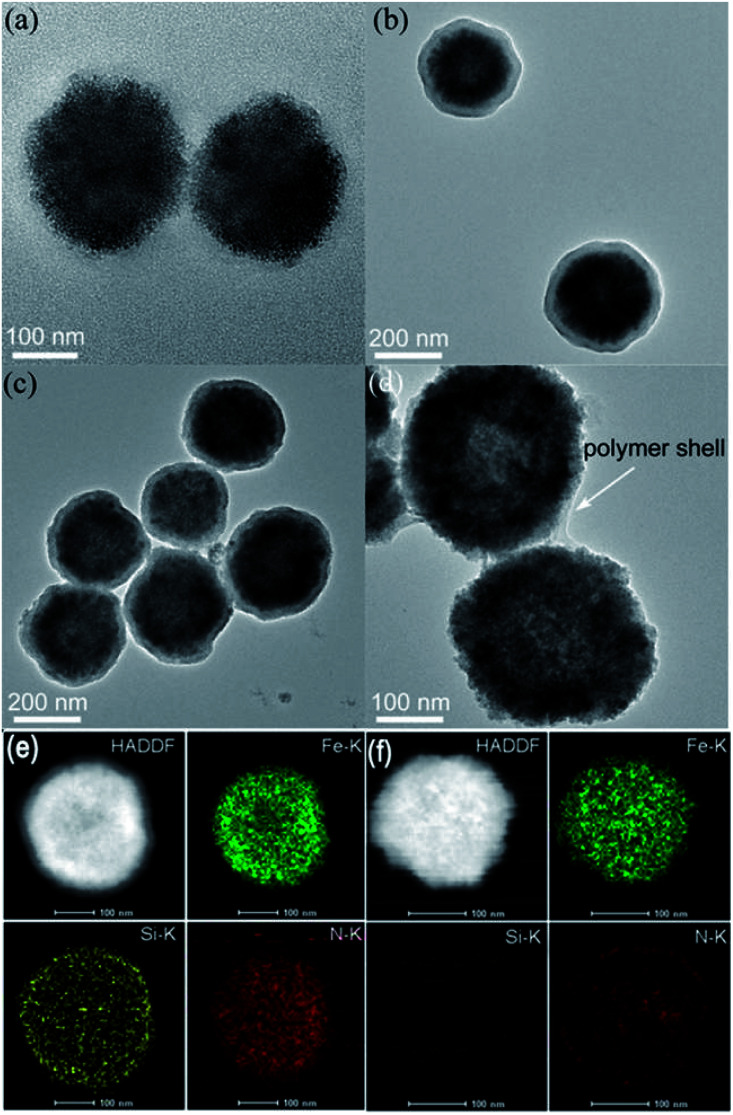
TEM images of (a) Fe_3_O_4_ NPs, (b) Fe_3_O_4_@SiO_2_, (c) Fe_3_O_4_@SiO_2_@Poly(l-Trp), and (d) yolk–shell Fe_3_O_4_@Poly(l-Trp). (e) and (f) show the TEM elemental mapping images of Fe_3_O_4_@SiO_2_@Poly(l-Trp) and yolk–shell Fe_3_O_4_@Poly(l-Trp), respectively.

Fe_3_O_4_ NPs were first prepared *via* a hydrothermal process by using citrate as a stabilizer. Then, Fe_3_O_4_ NPs were coated with a SiO_2_ shell and modified with 3-(trimethoxysilyl)propylmethacrylate (MPS) to introduce carbon–carbon double bonds. As shown in Fig. 1a and b, Fe_3_O_4_ NPs with an average diameter of *ca.* 200 nm were homogeneously coated with a SiO_2_ shell of about 25 nm thickness. Next, amino acid d-/l-tryptophan (Trp), d-/l-phenylalanine (Phe), d-/l-aspartic acid (Asp), and d-/l-histidine (His) appended polymer monomers were synthesized, respectively, and their corresponding ^1^H nuclear magnetic resonance (NMR) spectra were shown in the ESI.† Then Fe_3_O_4_@SiO_2_ was coated with different amino acid appended polymers. By taking the l-Trp appended polymer as an example, the formation of the polymer shell on the Fe_3_O_4_@SiO_2_ was first characterized by transmission electron microscopy (TEM). Obviously, after coating the polymer shell, the surface became rough (Fig. 1c). The thickness of the polymer shell was about 8.5 nm as determined by dynamic light scattering (DLS) at room temperature (Fig. S1†). The mid-layer silica of Fe_3_O_4_@SiO_2_@Poly(l-Trp) was further selectively removed by etching in a highly concentrated NaOH solution to form yolk–shell structured Fe_3_O_4_@Poly(l-Trp) nanoparticles. As shown in Fig. 1d, a thin polymer coating around the surface of Fe_3_O_4_ NPs was observed by TEM, which was consistent with the results of DLS determination (Fig. S1†). The TEM elemental mapping images shown in Fig. 1e were further used to confirm the preparation of yolk–shell structured Fe_3_O_4_@Poly(l-Trp). Silica and polymer shells in Fe_3_O_4_@SiO_2_@Poly(l-Trp) were confirmed by the representative elements Si and N. As shown in Fig. 1f, NaOH selective etching led to the disappearance of Si and retention of N, further confirming the formation of yolk–shell Fe_3_O_4_@Poly(l-Trp). The existence of the chiral polymer could also be supported by the obviously enhanced UV-vis absorption and circular dichroism (CD) spectra (Fig. S2 and S3†).The X-ray photoelectron spectroscopy (XPS) spectrum in Fig. S4† showed minimal interaction between Fe_3_O_4_ and the amino acid polymer, further confirming the successful synthesis of the yolk–shell Fe_3_O_4_@Poly(l-/d-Trp). 3,3,5,5-tetramethylbenzidine (TMB) was used to measure the catalytic activity of Fe_3_O_4_@Poly(AA) in the presence of H_2_O_2_. All the catalytic activities of Fe_3_O_4_@Poly(AA) were found to be similar to that of bare Fe_3_O_4_ NPs, as reported previously.^3^ Thus, yolk–shell Fe_3_O_4_@Poly(AA) nanoparticles still exhibit peroxidase activity (Fig. S5†). Notably, the core-shell-structured Fe_3_O_4_@SiO_2_@Poly(AA) showed no catalytic activity due to the blocking of the catalytically active site (data not shown), suggesting the necessity of a yolk–shell structure. Taking Fe_3_O_4_@Poly(l-Trp) as an example, it had peroxidase activity in a wide pH range (Fig. S5†).

**Fig. 2:**
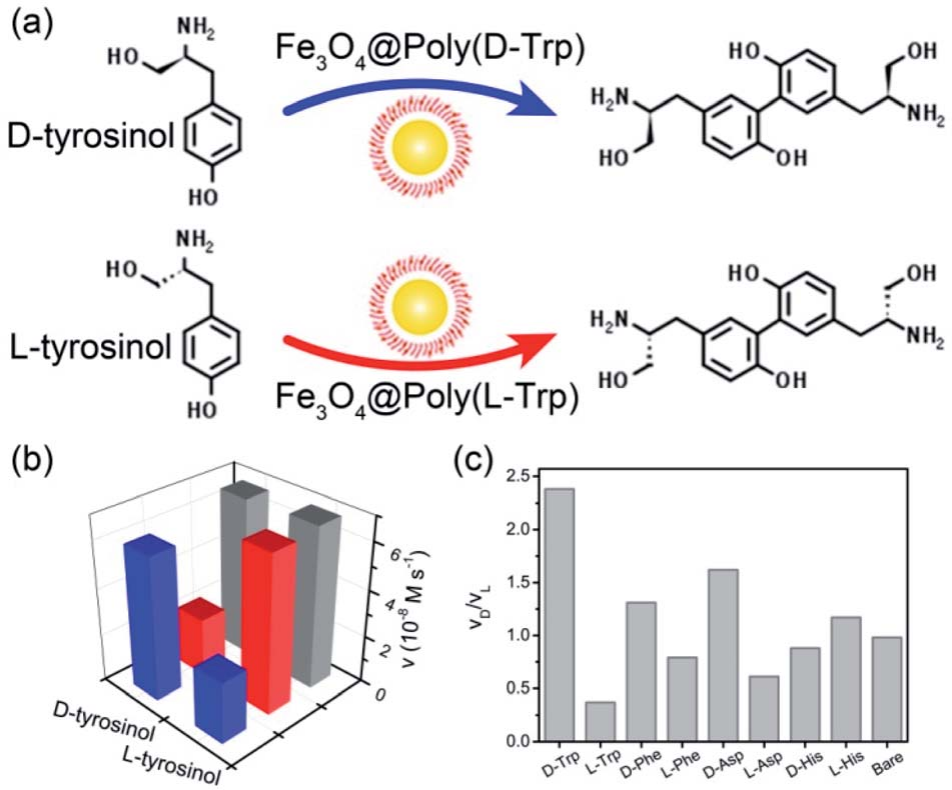
(a) The enantioselective oxidation of chiral tyrosinol catalyzed by Fe_3_O_4_@Poly(l-/d-Trp). (b) The initial velocity for the oxidation of tyrosinol enantiomers with Fe_3_O_4_@Poly(d-Trp) (blue), Fe_3_O_4_@Poly(l-Trp) (red) and bare Fe_3_O_4_ (gray). (c) The ratio of initial rates for the oxidation of the D enantiomer to L enantiomer.

In the following, the catalytic activities of Fe_3_O_4_@Poly(AA) toward a specific chiral substrate (l-/d-tyrosinol) were further studied by monitoring the time-dependent absorbance at 320 nm (Fig. 2a). The initial rates of the oxidation of l-/d-tyrosinol enantiomers could reveal the enantioselectivity of Fe_3_O_4_@Poly(AA). Fig. 2b showed the initial rates for the oxidation of l-and d-tyrosinol with Fe_3_O_4_@Poly(l-Trp), Fe_3_O_4_@Poly(d-Trp) and bare Fe_3_O_4_, respectively. Fe_3_O_4_@Poly(d-Trp) showed a better catalytic activity for d-tyrosinol than l-tyrosinol. The ratio calculated using the initial rates of oxidation of the d enantiomer and l enantiomer for Fe_3_O_4_@Poly(d-Trp) was 2.38. Interestingly, the enantioselectivity was reversed completely by using Fe_3_O_4_@Poly(l-Trp) with a ratio of 0.37, which was consistent with the first principles of stereochemistry.^16^ This indicated that the enantioselectivity of our designed nanozyme could be reversed readily by changing the chirality of the polymer shell. Similarly, the oxidation of d-/l-tyrosinol enantiomers by a series of Fe_3_O_4_@Poly(AA) was also investigated and the corresponding results were shown in Fig. 2c. As shown in Fig. 2c, Fe_3_O_4_@Poly(Phe) and Fe_3_O_4_@Poly(Asp) showed moderate enantioselectivity, while Fe_3_O_4_@Poly(His) showed poor enantioselectivity. In contrast, bare Fe_3_O_4_ did not show any stereoselectivity for oxidizing l-or d-tyrosinol.

To better understand the stereoselectivity of Fe_3_O_4_@Poly(Trp) towards tyrosinol enantiomers, we analyzed and compared the kinetic parameters based on the Michaelis–Menten model according to the saturation curves in Fig. S6.† The results were summarized in Table S1.† The smaller the *K*_M_, the higher the binding affinity between enzymes and substrates. The catalytic number (*k*_cat_) indicates the ability of enzymes to catalyze one certain substrate. *k*_cat_/*K*_M_ is usually applied for describing the catalytic efficiency of the enzyme. For Fe_3_O_4_@Poly(d-Trp), *k*_cat_ values for d- and l-tyrosinol were (16.44 ± 0.40)×10^3^ s^−1^ and (10.78 ± 1.27)×10^3^ s^−1^, respectively, indicating the higher catalytic ability of Fe_3_O_4_@Poly(d-Trp) for oxidizing d-tyrosinol rather than l-tyrosinol. Moreover, a lower *K*_M_ value was observed for d-tyrosinol than for l-tyrosinol, implying stronger binding affinity of Fe_3_O_4_@Poly(d-Trp) for d-tyrosinol. The higher catalytic activity and binding affinity to d-tyrosinol resulted in higher catalytic efficiency toward d-tyrosinol. As decided by *k*_cat_/*K*_M_ values, Fe_3_O_4_@Poly(d-Trp) was 5.38 times more active for d-tyrosinol than for l-tyrosinol. As for Fe_3_O_4_@Poly(l-Trp), the preference was reversed to l-tyrosinol due to the better catalytic ability and binding affinity to l-tyrosinol. Thus, the selectivity of the yolk–shell nanozyme originated from the higher activity and affinity to d-/l-tyrosinol and the preference was related to the chirality of the polymer. The selectivity factor of the yolk–shell nanozyme was higher than those of many chiral nanozymes reported before, such as graphene oxide-based peroxidase,^11*a*^ gold nanoparticle-based glucose oxidase,^11*b*^ gold nanoparticle-based phosphorylase,^11*c*^ ceria nanoparticle-based oxidase^11*d*^ and gold nanoparticle-based peroxidase^11*e*^ (Table S2†). In addition, in the oxidation of tyrosinol enantiomers, the Fe_3_O_4_@Poly(d-Trp) had a higher selectivity factor than HRP, which was reported to be 4.77 according to a previous report (Table S2†).^13*c*^ The improvement of enantioselectivity could be attributed to the strategies of the surface modifications.^9*b*,17^ Compared with the previously reported chiral nanozymes, in which the catalytic sites were partly covered by the direct modification of the chiral selector even though the density of the chiral selector was relatively low, our designed yolk–shell structure allowed highly dense modification of amino acids as the chiral selector but not direct covalent bonding to the central catalytic site. This enhanced the selectivity without blocking the catalytic site of the nanozyme.^15^ This design endowed the artificial peroxidase with high and on-demand enantioselectivity.

**Fig. 3:**
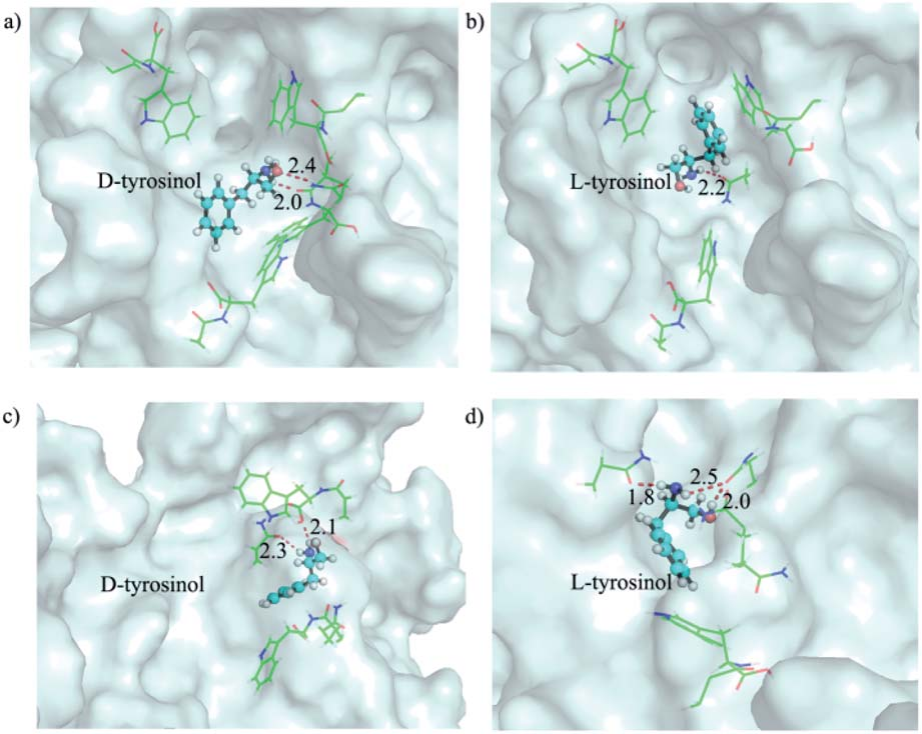
The representative binding models of d and l-tyrosinol on the outer surface of the (a, b) Poly(d-Trp) and (c, d) Poly(l-Trp) shell. The outer surfaces of Poly(d-/l-Trp) are shown as pale cyan surfaces. d-/l-tyrosinol is shown as a ball_and_stick model. The residues stabilizing d and l-tyrosinol by hydrophobic or hydrogen bond interactions are shown as lines. Hydrogen bonds are shown as red dashes and their distances are noted.

The selective binding affinity and catalytic activity of Fe_3_O_4_@Poly(AA) toward l-/d-tyrosinol were further confirmed. We first conducted dialysis experiments to reveal the binding preference.^18^ Fe_3_O_4_@Poly(AA) in the dialysis tube was dialyzed against racemic mixtures of tyrosinol, and circular dichroism was used to monitor the dialysate for enrichment of the enantiomer with weaker interaction with Fe_3_O_4_@Poly(AA). As shown in Fig. S7,† the dialysate for bare Fe_3_O_4_ was racemic, indicating no binding preference to tyrosinol enantiomers. The dialysate for Fe_3_O_4_@Poly(l-Trp) and Fe_3_O_4_@Poly(d-Trp) was rich in d-tyrosinol and l-tyrosinol, respectively, which obviously indicated the higher affinity of Fe_3_O_4_@Poly(l-Trp) to l-tyrosinol and Fe_3_O_4_@Poly(d-Trp) to d-tyrosinol. Here we proposed a possible catalytic process: d-/l-tyrosinol first diffused through the Poly(d-/l-Trp) shell, then reacted with the hydroxyl radicals, which were formed from the Fe_3_O_4_ NP-catalyzed decomposition of hydrogen peroxide. The obtained phenoxy radical could then couple with another d-/l-tyrosinol to give the final products. We supposed that the selectivity should be more relevant to the diffusion process of d-/l-tyrosinol since the bare Fe_3_O_4_ NPs displayed no selectivity to the tyrosinol enantiomers. The diffusion of l-/d-tyrosinol into the inner catalytic core through the Poly(d-/l-Trp) shell could be divided into three processes: adsorption, transport and desorption. We then examined the role of l-/d-tyrosinol adsorption/desorption using a patch of Poly(d-/l-Trp) shell models by molecular simulation methods (Fig. S8†). The amino acid-appended polymer shell could selectively transport l-/d-tyrosinol from the outer solution into the inner catalytic yolk. Therefore, the adsorption of l-/d-tyrosinol on the outer surface of the Poly(d-/l-Trp) shell and the desorption from the inner surface of the Poly(d-/l-Trp) shell could give information about the binding preference. In order to clearly demonstrate this fact, we investigated the top 3 binding structures of l-/d-tyrosinol with the outer and inner surface of Poly(d-/l-Trp) (shown in Fig. S9†), respectively. The detailed illustration in Fig. 3 depicted the best binding mode of l-/d-tyrosinol on the outer surface of the Poly(d-/l-Trp) shell. We also examined their corresponding average adsorption free energy (AFE) on the outer surface and desorption free energy (DFE) from the inner surface of Poly(d-/l-Trp) (listed in Table S3†) based on the top 3 binding models. It was found that the AFE of d-tyrosinol on the outer surface of Poly(d-Trp) was much lower than that of l-tyrosinol by 2.9 kcal mol^−1^, indicating that Poly(d-Trp) was more prone to adsorb d-tyrosinol, not l-tyrosinol. Similarly, Poly(l-Trp) was more favourable for the adsorption of l-tyrosinol with a free energy difference of 1.8 kcal mol^−1^ compared to d-tyrosinol. More interestingly, we also noted that the desorption of d-tyrosinol from Poly(d-Trp) and l-tyrosinol from Poly(l-Trp) was easier than the opposite configuration because of the lower binding free energy. Nevertheless, it was also found that the differences of adsorption free energy were distinctly larger than those of desorption free energy, indicating that the adsorption process played an important role in the enantioselectivity of Poly(d-/l-Trp). Furthermore, we also decomposed the binding free energy into individual energy components to shed light on the dominant interaction for driving d-tyrosinol and l-tyrosinol binding to Poly(d-/l-Trp), as presented in Table S4.† We found that van der Waals interactions provided a major contribution to the difference of adsorption free energy of d-tyrosinol and l-tyrosinol on the outer surface of Poly(d-Trp) and Poly(l-Trp). This indicated that van der Waals interactions were closely correlated with the enantioselectivity of Fe_3_O_4_@Poly(d-/l-Trp). Electrostatic interactions, determined by the hydrogen bond interactions, were favourable for the adsorption of d-tyrosinol on the outer surface of Poly(d-Trp), while it showed unfavourable contributions to the adsorption of l-tyrosinol on the outer surface of Poly(l-Trp). The results of molecular simulation were consistent with dialysis experiments, and demonstrated that Poly(d-Trp) and Poly(l-Trp) contributed to the binding preference of Fe_3_O_4_@Poly(d-/l-Trp) to d-/l-tyrosinol. It is worth mentioning that the transport dynamics of l-/d-tyrosinol within the Poly(d-/l-Trp) shell should also play an important role in the catalytic enantioselectivity, which will be studied in the future. Then, the activation energy (*E*_a_) was calculated according to the Arrhenius equation^19^ (Fig. S10†). For Fe_3_O_4_@Poly(d-Trp), the *E*_a_ values for d-tyrosinol and l-tyrosinol were estimated to be 65.1 ± 2.3 kJ mol^−1^ and 86.6 ± 2.4 kJ mol^−1^, respectively. It was obvious that in the presence of Fe_3_O_4_@Poly(d-Trp), the energy barrier for the oxidation of d-tyrosinol was lower. In contrast, Fe_3_O_4_@Poly(l-Trp) exhibited a lower energy barrier for the oxidation of l-tyrosinol. The results confirmed undoubtedly the higher catalytic activity of Fe_3_O_4_@Poly(d-Trp) to d-tryosinol and Fe_3_O_4_@Poly(l-Trp) to l-tyrosinol, which was consistent with the kinetic parameters.

The performance of Fe_3_O_4_@Poly(l-/d-Trp) in enantioselective catalysis was further studied by high-performance liquid chromatography (HPLC). As shown in Fig. S11,† in the presence of Fe_3_O_4_@Poly(l-Trp), l-tyrosinol was almost consumed to generate the product with an 89% yield. The main product was found to be l-dityrosinol according to the ^1^H NMR spectra in Fig. S12a.† In contrast, the yield for the reaction of d-tyrosinol was just 32%. For Fe_3_O_4_@Poly(d-Trp), d-tyrosinol was almost transformed to d-dityrosinol with a 92% yield as shown in Fig. S11d and S12b,† while the yield for the reaction of l-tyrosinol was only 29%. The results displayed the catalytic selectivity of our nanozymes toward l-/d-tyrosinol. The chiral nanozyme was then applied for the oxidation of racemic tyrosinol. As shown in Fig. S13,† with Fe_3_O_4_@Poly(l-Trp), l-tyrosinol was almost consumed while d-tyrosinol was in excess, indicating the higher conversion of l-tyrosinol. As for Fe_3_O_4_@Poly(d-Trp), d-tyrosinol was almost consumed while l-tyrosinol was in excess, indicating the higher conversion of d-tyrosinol. The oxidation of the racemic substrate further proved that the oxidation of tyrosinol by Fe_3_O_4_@Poly(l-/d-Trp) was enantioselective. Besides, the enantiomeric excess was up to 99% toward d-tyrosinol with the catalysis of Fe_3_O_4_@Poly(l-Trp). This meant that enantiopure d-tyrosinol was prepared. Similarly, enantiopure l-tyrosinol was prepared from racemate tyrosinol with the catalysis of Fe_3_O_4_@Poly(d-Trp). Significantly, the results suggested the potential ability of our artificial peroxidase for preparation of optically pure compounds.

**Fig. 4:**
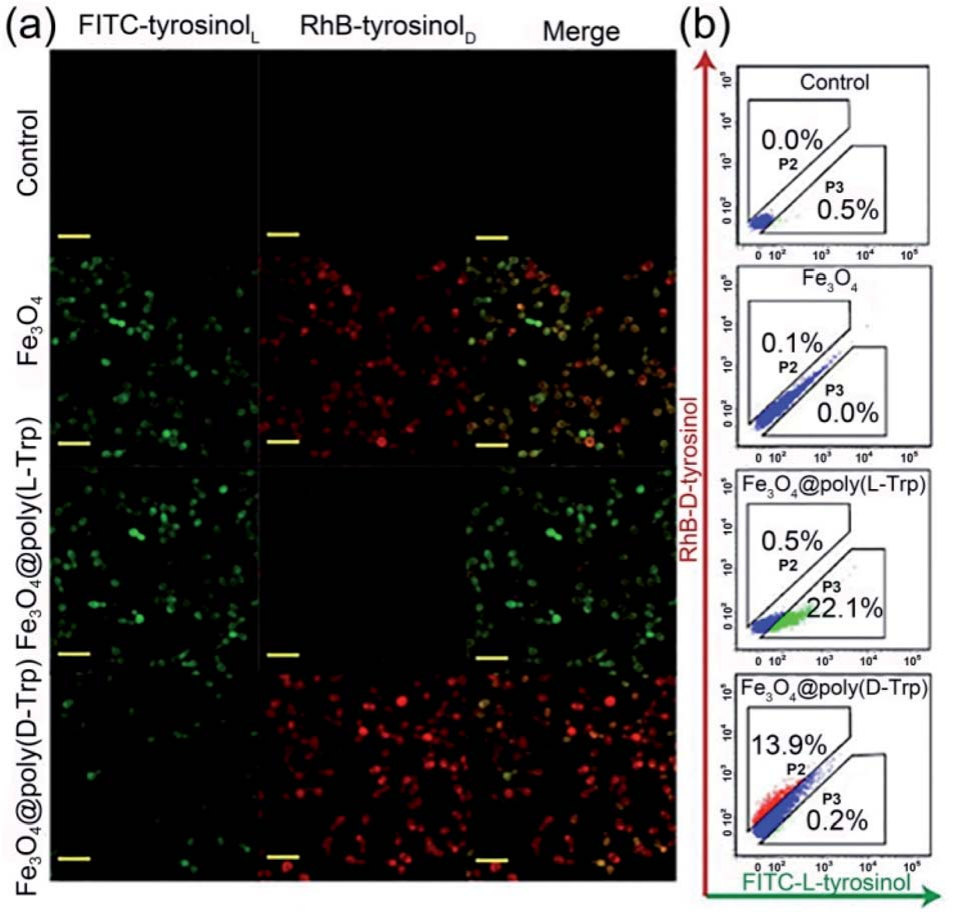
(a) Confocal fluorescence microscopy and (b) flow cytometry analysis results of yeast cells treated with H_2_O_2_ (100 μM), FITC-tyrosinol_L_ and RhB-tyrosinol_D_ with no nanozyme (control), bare Fe_3_O_4_, Fe_3_O_4_@Poly(l-Trp) or Fe_3_O_4_@Poly(d-Trp), respectively. (Scale bars: 20 μm.)

Then, we verified whether the catalytic activity and selectivity toward l-/d-tyrosinol of our designed nanozymes can be realized in living cells. Live/dead cell staining was first performed. As shown in Fig. S14,† the nanozyme and H_2_O_2_ showed minimal influence on the cell viability. Klibanov *et al.* demonstrated that reactive oxygen species can catalyze tyrosinol to form free phenol radicals, subsequently covalently linked to the tyrosine residues on the surface of yeast cells.^20^ For visualizing these processes, we modified the l-/d-tyrosinol with FITC and Rhodamine B (RhB), marked as FITC-tyrosinol_L_ and RhB-tyrosinol_D_. Yeast cells were treated with FITC-tyrosinol_L_ and RhB-tyrosinol_D_ in the presence of bare Fe_3_O_4_, Fe_3_O_4_@Poly(l-Trp) or Fe_3_O_4_@Poly(d-Trp), respectively (Scheme S1†). As shown in Fig. 4, for the control group, negligible fluorescence was observed in the yeast cells. For the bare Fe_3_O_4_ group, both green and red fluorescence were observed, which suggested that bare Fe_3_O_4_ NPs had no enantioselectivity towards tyrosinol. For the Fe_3_O_4_@Poly(l-Trp) group, the green fluorescence was much stronger than the red fluorescence, indicating the high enantioselectivity of Fe_3_O_4_@Poly(l-Trp) toward l-tyrosinol. For the Fe_3_O_4_@Poly(d-Trp) group, strong red fluorescence was observed on the surface of yeast cells, indicating the high enantioselectivity of Fe_3_O_4_@Poly(d-Trp) towards d-tyrosinol. Flow cytometry (Fig. 4b) also showed similar results, further supporting the confocal fluorescence data.

**Fig. 5:**
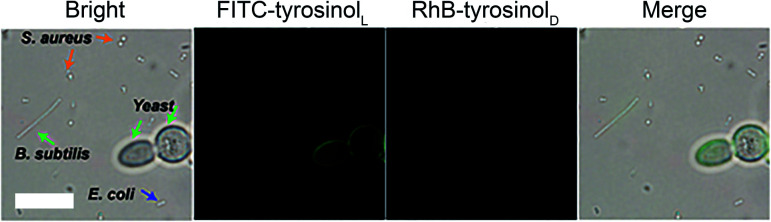
Fe_3_O_4_@Poly(l-Trp) NPs catalyze FITC-l-tyrosinol to label yeast cell with specificity. Fluorescence images of different microbial cells (yeast, *S.aureus*, *E.coli* and *B.subtilis*) after treatment with Fe_3_O_4_@Poly (l-Trp), H_2_O_2_ (100 μM), FITC-tyrosinol_L_ and RhB-tyrosinol_D_. (Scale bars: 10 μm.)

We also attempted to label bacterial cells, such as *S.aureus*, *E.coli* and *B.subtilis* with FITC-tyrosinol_L_/RhB-tyrosinol_D_ by using the same assay as we did for yeast cells. As shown in Fig. S15–20,† neither green emission nor red emission was observed in all groups including the control, bare Fe_3_O_4_, Fe_3_O_4_@Poly(l-Trp) and Fe_3_O_4_@Poly(d-Trp). These results indicated that the surface of *S.aureus*, *E.coli* and *B.subtilis* cells were difficult to label with a tyrosinol-based fluorescent agent. Next four different microbial cells (yeast, *S.aureus*, *E.coli* and *B.subtilis*) were treated with Fe_3_O_4_@poly(l-Trp), H_2_O_2_, FITC-tyrosinol_L_ and RhB-tyrosinol_D_ to demonstrate the specificity of the labelling process towards yeast. As shown in Fig. 5, only yeast cells showed green fluorescence among these cells because of the abundant tyrosine residues on the surface of yeast cells.^20^ The different labelling results on *S.aureus*, *E.coli* and *B.subtilis* might be due to the different structures and chemical compositions of their cell walls. The specific feature of the Gram-positive bacterial cell wall (*S. aureus*) is that it is composed of teichoic/lipoteichoic acids. The characteristic components of the Gram-negative bacterial cell wall (*E.coli* and *B.subtilis*) are lipopolysaccharides.^21^ Fewer tyrosine residues exist on the bacterial cell wall compared with the yeast cells. Thus fewer fluorescent agents could be labelled on their surface.

## Conclusions

In summary, we present an unprecedented approach to integrate the active yolk artificial peroxidase Fe_3_O_4_ NP and d- or l-amino acid-appended chiral selective polymer shell for construction of a series of yolk–shell structured artificial peroxidases. Different from the chiral nanozymes with direct surface modification, the chiral artificial peroxidases exhibit high enantioselectivity. Among them, Fe_3_O_4_@Poly(d-Trp) shows the highest selectivity factor of 5.38, which is even higher than that of HRP. The enantioselectivity is readily reversed by replacing d-Trp with l-Trp. Kinetic parameters, dialysis experiments, and molecular simulations together with activation energy reveal that the d-/l-Trp appended polymer shell results in better affinity and catalytic activity to d-/l-tyrosinol, thus leading to the selectivity of Fe_3_O_4_@Poly(d-Trp) to d-tryosinol and Fe_3_O_4_@Poly(l-Trp) to l-tryosinol, respectively. These artificial peroxidases could be used for preparing enantiopure d- or l-enantiomers. Besides, confocal fluorescence microscopy and flow cytometry analysis have demonstrated that fluorescent dye-modified chiral tyrosinol can selectively label yeast cells among *S.aureus*, yeast, *E.coli* and *B.subtilis* in the presence of our designed nanozyme. This work would promote rational design and synthesis of stereoselective artificial enzymes.

## Conflicts of interest

There are no conflicts to declare.

## Supplementary Material

SC-011-D0SC03082A-s001

## References

[cit1] Huang X., Liu X., Luo Q., Liu J., Shen J. (2011). Chem. Soc. Rev..

[cit2] Manea F., Houillon F. B., Pasquato L., Scrimin P. (2004). Angew. Chem., Int. Ed..

[cit3] Gao L., Zhuang J., Nie L., Zhang J., Zhang Y., Gu N., Wang T., Feng J., Yang D., Perrett S., Yan X. (2007). Nat. Nanotechnol..

[cit4] Song Y., Qu K., Zhao C., Ren J., Qu X. (2010). Adv. Mater..

[cit5] Huo M., Wang L., Chen Y., Shi J. (2017). Nat. Commun..

[cit6] Natalio F., Andre R., Hartog A. F., Stoll B., Jochum K. P., Wever R., Tremel W. (2012). Nat. Nanotechnol..

[cit7] Li Y., He X., Yin J. J., Ma Y., Zhang P., Li J., Ding Y., Zhang J., Zhao Y., Chai Z., Zhang Z. (2015). Angew. Chem., Int. Ed..

[cit8] Liu X., Wang Q., Zhao H., Zhang L., Su Y., Lv Y. (2012). Analyst.

[cit9] Fan K., Wang H., Xi J., Liu Q., Meng X., Duan D., Gao L., Yan X. (2016). Chem. Commun..

[cit10] Ghosh S., Roy P., Karmodak N., Jemmis E. D., Mugesh G. (2018). Angew. Chem., Int. Ed..

[cit11] Xu C., Zhao C., Li M., Wu L., Ren J., Qu X. (2014). Small.

[cit12] Mallat T., Orglmeister E., Baiker A. (2007). Chem. Rev..

[cit13] Deurzen M., Rantwijk F., Sheldon R. A. (1997). Tetrahedron.

[cit14] Gross E., Toste F. D., Somorjai G. A. (2015). Catal. Lett..

[cit15] Sakaki K. , in Encyclopedia of Membranes, ed. E. Drioli and L. Giorno, Springer Berlin Heidelberg, Berlin, Heidelberg, 2015, pp. 1–2, 10.1007/978-3-642-40872-4_1428-3

[cit16] Shabbir S. H., Regan C. J., Anslyn E. V. (2009). Proc. Natl. Acad. Sci. U. S. A..

[cit17] Rossi N. A., Constantinescu I., Brooks D. E., Scott M. D., Kizhakkedathu J. N. (2010). J. Am. Chem. Soc..

[cit18] Li M., Howson S. E., Dong K., Gao N., Ren J., Scott P., Qu X. (2014). J. Am. Chem. Soc..

[cit19] Sun H., Zhao A., Gao N., Li K., Ren J., Qu X. (2015). Angew. Chem., Int. Ed..

[cit20] Lipovsek D., Antipov E., Armstrong K. A., Olsen M. J., Klibanov A. M., Tidor B., Wittrup K. D. (2007). Chem. Biol..

[cit21] Bugla-Ploskonska G. (2008). Postępy Mikrobiologii.

