# Supplementary material for: Construction of a chiral artificial enzyme used for enantioselective catalysis in live cells
Source: Chem Sci. 2020 Sep 23;11(41):11344–50. doi: 10.1039/d0sc03082a (PMC8162767; doi:10.1039/d0sc03082a)
Supplement: SC-011-D0SC03082A-s001 [file SC-011-D0SC03082A-s001.pdf]

Electronic Supplementary Material (ESI) for Chemical Science.
This journal is © The Royal Society of Chemistry 2020

Supporting Information

Construction of a chiral artificial enzyme used for enantioselective catalysis in live cells

Ya Zhou,[a,b] Weili Wei,[a] Fengchao Cui,[a,b] Zhengqing Yan,[a] Yuhuan Sun,[a,b] Jinsong Ren,[a,b] and Xiaogang Qu*[a,b]

[a]Laboratory of Chemical Biology and State Key Laboratory of Rare Earth Resource Utilization, Changchun Institute of Applied Chemistry, Chinese Academy of Sciences, Changchun, Jilin 130022, China.

[b]University of Science and Technology of China, Hefei, Anhui 230026, China.

Methods

1. Apparatus and characterization

UV absorbance measurements were carried out on a JASCO V-550 UV-vis spectrophotometer, equipped with a Peltier temperature control accessory. TEM images were recorded using a FEI TECNAI G2 20 high-resolution transmission electron microscope operating at 200 kV. Dynamic light scattering (DLS) measurements were performed on a Nano-ZS Zetzsozer ZEN3600 instrument (Malvern Instruments Ltd., UK). The flow cytometry data were recorded using BD LSRFortessa™ cell analyzer.

2. Synthesis and surface modification of Fe_3O_4@SiO_2

Magnetite particles were synthesized as follows. 3.0 g of $FeCl_3$ $6H_2O$, 0.72 g of trisodium citrate and 4.8 g of sodium acetate were dissolved in 100 mL of ethylene glycol under vigorous stirring for 30 min. The resultant mixture was then transferred into a Teflon-lined stainless-steel autoclave (with a capacity of 200 mL) for heating at 200 ℃ for 10 h. After that, the autoclave was carefully taken out to cool to room temperature. The as-made black products were thoroughly washed with ethanol and deioned water for several times, and

finally vacuum dried at 25 ℃. To synthesize Fe_3O_4@SiO_2 microspheres, 50 mg of magnetite particles were fully dispersed in a solution containing 160 mL of ethanol and 40 ml of H_2O 2.0 mL of concentrated aqua ammonia (28 wt%) under ultrasonication vibration. Then, 1.0 ml of tetraorthosilicate (TEOS) was added by injection to the resultant basic dispersion, followed by mechanically stirring for 6 h at 30 ℃. The obtained Fe_3O_4@SiO_2 microspheres were washed with ethanol for several times to remove blank silica nanoparticles. In order to modify the surface of Fe_3O_4@SiO_2 microspheres with MPS silane coupling agent, the purified Fe_3O_4@SiO_2 microspheres were redispersed in 80 mL of ethanol and 0.5 mL of MPS was added to the dispersion. After mechanically stirred for 48 h at 30 ℃, the Fe_3O_4@SiO_2-MPS microspheres were washed with ethanol with the help of a magnet, and redispersed in 50 mL of ethanol for further use.

3. Synthesis of chiral monomers

The synthesis of N-Acryloyl-amino acids were performed following the procedure. Take Phe as the example. 3.0 g of L-phenylalanine was dissolved in a well-stirred aqueous solution (15 mL) of sodium hydroxide (0.8 g). Acryloyl chloride (1.5 mL) was added drop-wise over a 30 min period. The reaction was kept at a temperature below 0 ℃ by cooling in an ice bath. Then, the stirred solution was acidified to pH 1 with HCl. The precipitate was filtered and recrystallized from water (yield: 76%).

4. Synthesis of Fe_3O_4@SiO_2@Ploy(AA)

The sandwich structured Fe_3O_4@SiO_2@Poly(AA) microspheres were synthesized via an aqueous-phase radical polymerization. Typically, in a three-necked round bottom flask (100 mL) equipped with a mechanical stirrer, a refluxing condenser, and a nitrogen inlet, 10 mL of ethanol dispersion of Fe_3O_4@SiO_2-MPS microspheres were mixed with 50 mL of aqueous solution containing 5.0 mg of sodium lauryl benzenesulfate under mechanically stirring. After stirring for 1h, 1.0 g of polymer monomers (acrylamide:chiral amino acid monomers=4:1) and 72.3 mg of bis-acrylamide were added, and the resultant dispersion was bubbled with high-

purity nitrogen gas for 0.5 h. Then, the flask was immerged into a preheated oil bath at 70 ℃ and the polymerization was carried out with a stirring speed of 200 rpm at 70 ℃ for 24 h. The resulting Fe_3O_4@SiO_2@Poly(AA) were harvested and washed by magnetic separation.

5. Synthesis of yolk-shell-structured Fe_3O_4@Ploy(AA)

Etching of the silica layer of the Fe_3O_4@SiO_2@Poly(AA): Fe_3O_4@Poly(AA) yolk–shell particles were prepared by a silica-etching procedure: NaOH (1 mL, 2M) solution was added into a Fe_3O_4@SiO_2@Poly(AA) suspension (4 mL, 1 mg NPs). The mixture was gently stirred at room temperature overnight and was cleaned by a magnet.

6. Bioassay

Kinetic measurements were carried out in time course mode by monitoring the absorbance change of tyrosinol at 320 nm. Experiments were carried out using 250 μg mL^{-1} nanozymes in a reaction volume of 500 μL buffer solution (25 mM Na_2HPO_4, pH 4.0, 37 ℃) with tyrosinol as substrate, and H_2O_2 concentration was 50 mM, unless otherwise stated. Accordingly, the oxidation rates of tyrosinol in the presence of the different nanozymes were analyzed in terms of the Michaelis-Menten model: $\nu = V_{max}C/(K_m+C)$, where ν is the initial velocity, V_{max} is the maximal reaction velocity, and C is the concentration of substrate. k_{cat} was calculated according to the equation: $k_{cat} = V_{max}/S$, where S is the concentration of catalyst.[1]

7. Synthesis of FITC-tyrosinol$_L$ and RhB-tyrosinol$_D$

A solution of FITC (195mg, 0.5 mmol) or Rhodamine B (RhB) (240mg, 0.5 mmol), 4-DMAP (43 mg, 0.35 mmol), and EDC·HCl (115 mg, 0.6 mmol) in DMF (20 ml) was incubated at room temperature for 30 min and then added dropwise to a solution of L- or D-tyrosinol·HCl (204 mg, 1.0 mmol) and triethylamine (2.5 mmol) in 10 ml of DMF. The resulting reaction mixture was stirred overnight at room temperature, evaporated, and redissolved in 20 ml of 0.1 M HCl. The mixture was extracted with ethyl acetate (3×60 ml), washed with saturated aqueous $NaHCO_3$ (3×60 ml) and with brine (60 ml), dried over

anhydrous Na_2SO_4 and evaporated to give a crude product that was then purified by column chromatography.[2]

8. Cell labelling

Microbial cells treated with 100 μg mL^{-1} Fe_3O_4@poly(L-/D-Trp), H_2O_2(100 μM), FITC-tyrosinol$_L$ (20 μg mL^{-1}), and RhB-tyrosinol$_D$ (12 μg mL^{-1}) and the mixture was incubated at 37 ℃ for 30 min. The cells were washed twice with PBS solution, the fluorescence intensity was monitored by flow cytometric analysis. Fluorescence images were captured with an Olympus BX-51 optical equipped with a CCD camera.

9. Characterizations of cell labelling by confocal laser scanning microscopy (CLSM)

Cells treated with different agents were incubated for 30 min. After rinsed with PBS buffer to remove excess dye, the cells were observed by CLSM. Then images were acquired using a (Nikon Eclipse Ni-E, Japan) top-of-the-line motorized upright CLSM. All samples were observed using the same parameters and analyzed utilizing NIS-Elements software.

10. HPLC study

After reacted under 37 ℃ for 12 h, the products was analyzed on a MCI GEL CRS10W reverse phase column (Mitsubishi Chemical Corporation) eluting with 0.005 M $CuSO_4$.

11. Computational details

11.1 Constructed protocols of Poly(AA) shell

The cross-linked Poly(AA) is composed of acrylamide (AM) and N-Acryloyl-amino acids (NA-AA) as monomers and N,N'-Methylenebisacrylamide (MBA) as cross-linker. The single repeating unit, MBA and the specific chiral substrate (L-/D-tyrosinol) were firstly built by GaussView and optimized with B3LYP/6-31G(d, p) using Gaussian 09 program[7]. The non-crosslinking Poly(AA) melt was generated by packing 25 linear chains in which each chain was constructed with 49 AM monomers and 16 NA-AA monomers (the approximate ratio 4:1). Then Poly(AA) chains were cross-linked artificially with MBAs ((AM+NA-AA)/MBA = 30:1) randomly inserted into the free volume of Poly(AA) matrix. Atomic interactions was

described by general AMBER force field (GAFF)[8], and R.E.D. tools[9] with restrained electrostatic potential (RESP) approaches were used to obtain the partial charges. Taking D/L-Trp appended Poly(AA) as an example, the constructed cross-linked Poly(AA) networks were firstly minimized with conjugate gradient minimization of 10000 steps, followed by 1 ns MD simulations under NPT ensemble. Then, the annealing process (from 300 to 800 K and back) was performed to relax the cross-linked Poly(AA) networks to the approximate global minimum with NPT ensmble. The cross-linked Poly(AA) networks were further equilibrated about 20 ns at 300 K and 1 atm under NPT ensemble.

11.2 Complex structures of Poly(L-Trp/D-Trp) and L-/D-tyrosinol

Molecular dockings of L-/D-tyrosinol to Poly(L-Trp/D-Trp) were respectively carried out by AutoDock Vina,[10] in which the Iterated Local Search Globule Optimizer[11] was applied to locate the most favorable binding site. Semi-flexible docking method was used, where Poly(L-Trp/D-Trp) was treated as a rigid body and all rotatable bonds in L-/D-tyrosinol were sampled. Optimal binding sites were searched in a box of $30\times84\times61$ Å^3 which covered the inner and outer surface of Poly(L-Trp/D-Trp) shell. The box had 1.0 Å grid spacing and centered at the geometric center of the terminal residues of inner and outer surface. In each docking experiment, top structure model in three different binding sites were selected according to the binding affinity calculated by the scoring function in AutoDock Vina.

11.3 Adsorption and desorption free energy calculation

Three optimal complex structure models from docking for each case were further refined in a fully flexible atomic molecular dynamics simulations where conjugate gradient minimization of 5000 steps and 20 ns equilibrations were performed under NPT ensemble with 300 K. In each simulation trajectory, 500 complex structure models at a time interval of 40 ps were extracted and used to calculate adsorption free energy calculation using the molecular mechanics Poisson-Boltzmann solvent accessible surface area (MM-PBSA) method[12]. The total binding free energy can be calculated by

$$\Delta G_{bind} = \Delta E_{MM} + \Delta G_{sol} - T\Delta S$$
$$= (\Delta E_{vdW} + \Delta E_{ele} + \Delta E_{int}) + (\Delta G_{polar} + \Delta G_{nonpolar}) - T\Delta S$$

(4)

here, ΔE_{MM} is the change of molecular mechanical energy without considering solvent, ΔG_{sol} is the solvation free energy and $T\Delta S$ considers the penalty of conformational entropy. ΔE_{MM} as non-bonded interaction includes van der Waals ΔE_{vdW} and electrostatic ΔE_{ele} interactions, and local interaction ΔE_{int} with energy terms of bond, angle, and dihedral. ΔE_{int} was counteracted in the single MD trajectory approach. The solvation free energy ΔG_{sol} is the sum of electrostatic/polar (ΔG_{polar}) and nonpolar ($\Delta G_{nonpolar}$) contributions, i.e.,

$$\Delta G_{sol} = \Delta G_{polar} + \Delta G_{nonpolar} \quad (5)$$

ΔE_{MM} was calculated using Amber 12[13] without cutoff treatment for nonbond energies. ΔG_{polar} was estimated by the PB numerical solver implemented in the PBSA module in AmberTools 13[14]. In the MM-PBSA calculations, a grid spacing of 0.5 Å is employed for the cubic lattices, and relative dielectric constant was set to 80.0 at the exterior and 2.0 at the interior of catechin-trypsin complex. $\Delta G_{enpolar}$ was calculated by solvent accessible surface area ($\Delta G_{enpolar}=\gamma A+b$) dependent term of γ coefficient of 0.0378 kcal/molÅ^2 and b offset of -0.5692 kcal/mol with a solvent-probe radius of 1.4 Å. Considering the low prediction accuracy and computationally time-comsuming[15], we ignored the entropy contributions in the present study.

All MD simulations were carried out under NPT ensemble in the present study. Langevin thermostat was used to maintain the temperature at 300 K with the dampening coefficient of 5 ps^{-}. Pressure was scaled at 1 atm with Nosé-Hoover Langevin piston method [16] with the piston period of 100 fs, the piston decay of 50 fs, and the piston temperature at 300 K. Periodic boundary conditions were applied and long-range electrostatics were treated using the particle-mesh Ewald (PME) method.[17] Non-bonded interactions were calculated using a cutoff of 12 Å without switch function, and a 14 Å neighbor list was updated every 10 steps

of the dynamics. The integrated time step is set to 2 fs. All covalent bonds involving hydrogen atoms were confined by SHAKE algorithm.[18] All MD simulations were performed with NAMD 2.9 program.[19]

Table S1. Kinetic parameters for catalytic oxidation of tyrosinol enantiomers by Fe_3O_4@Poly(D-Trp) or Fe_3O_4@Poly(L-Trp).

Artificial peroxidises[a]	Substrate	$K_M(10^{-3}M)$	$k_{cat}(10^3 s^{-1})$	$k_{cat}/K_M(10^{-6}M^{-1}s^{-1})$	Selectivity Factor[b]
Fe_3O_4@Poly(D-Trp)	D-tyrosinol<br>L-tyrosinol	6.49±0.86<br>22.73±2.46	16.44±0.40<br>10.78±1.27	2.53<br>0.47	5.38
Fe_3O_4@Poly(L-Trp)	D-tyrosinol<br>L-tyrosinol	14.08±0.96<br>6.04±0.82	8.64±0.43<br>14.8±0.85	0.61<br>2.45	4.02

[a]The concentration of nanozymes was calculated on the basis of the total mass of Fe_3O_4.The Fe content in these mimetic enzymes was determined by ICP analysis.

[b] Selectivity factor of Fe_3O_4@Poly(L-Trp) equals $[k_{cat}/K_M]$L-tyrosinol/$[k_{cat}/K_M]$D-tyrosinol. Selectivity factor of Fe_3O_4@Poly(D-Trp) equals $[k_{cat}/K_M]$D-tyrosinol/$[k_{cat}/K_M]$L-tyrosinol. Each standard deviation was calculated from three measurements.

Table S2. The selectivity factors of current reported chiral nanozymes and HRP.

Chiral Nanozymes and References	Substrate	Selectivity Factor (SF)[d]
Fe_3O_4@Poly(D-Trp) peroxidases	D-tyrosinol	5.38
Graphene oxide-based peroxidase [3]	TMB[a]	1.27
Gold NP-based glucose oxidase[4]	D-glucose	1.84
Gold NP-based phosphorylase[5]	UpU[b]	4.00
Ceria NP-based Oxidase[1b]	D-DOPA[c]	1.87
Gold NP-based peroxidase[6]	D-DOPA	1.69
HRP	L-Tyrosinol	4.77

[a-c)] TMB, UpU and DOPA represent 3,3',5,5'-Tetramethylbenzidine; dinucleotides, 3,4-dihydroxyphenylalanine respectively.

[d)] The SF values were achieved from the published works.

Table S3. The adsorption/desorption free energy (AFE and DFE) of tyrosinol enantiomers on the outer surface/from inner surface of Poly(D-Trp) or Poly(L-Trp) shell and their corresponding Boltzmann Factor.

Shell	Substrate	AFE (kcal/mol)	Boltzmann Factor[a]	DFE (kcal/mol)	Boltzmann Factor[a]
Poly(D-Trp)	D-tyrosinol<br>L-tyrosinol	-19.4±1.9<br>-16.5±4.0	110.51	-21.1±2.5<br>-21.4±2.1	0.59
Poly(L-Trp)	L-tyrosinol<br>D-tyrosinol	-22.9±1.0<br>-21.1±3.5	18.55	-19.8±3.3<br>-20.4±3.4	0.40

[a] The Boltzmann factor tells us the relative probability with which D-tyrosinol and L-tyrosinol adsorb on the outer surface or desorb from the inner surface of Poly(D-Trp) or Poly(L-Trp) at catalytic temperature 37 ℃.

Table S4. The adsorption and desorption free energies (AFE and DFE) and their components for D-/L-tyrosinol binding on the outer or inner surface of Poly(D-/L-Trp) shell, respectively.

Component	Poly(D-Trp)				Poly(L-Trp)			
	D-tyrosinol		L-tyrosinol		D-tyrosinol		L-tyrosinol	
	outer	inner	outer	inner	outer	inner	outer	inner
ΔE_{vdw}	-18.1±1.9	-20.7±2.8	-16.1±4.7	-20.6±2.3	-21.4±4.2	-19.6±3.8	-23.5±1.9	-18.5±4.2
ΔE_{ele}	-5.0±1.2	-3.7±0.9	-3.2±1.5	-3.6±2.1	-3.7±1.2	-4.8±1.1	-3.0±1.9	-4.8±1.2
ΔG_{polar}	6.0±0.7	5.7±0.8	4.9±0.7	5.2±1.2	6.2±0.9	6.3±0.7	5.9±0.8	5.8±0.4
$\Delta G_{nonpolar}$	-2.3±0.2	-2.4±0.1	-2.1±0.1	-2.4±0.2	-2.2±0.2	-2.3±0.2	-2.3±0.1	-2.3±0.1
$\Delta G_{AFE/DFE}$	-19.4±1.9	-21.1±2.5	-16.5±4.0	-21.4±2.1	-21.1±3.5	-20.4±3.4	-22.9±1.0	-19.8±3.3

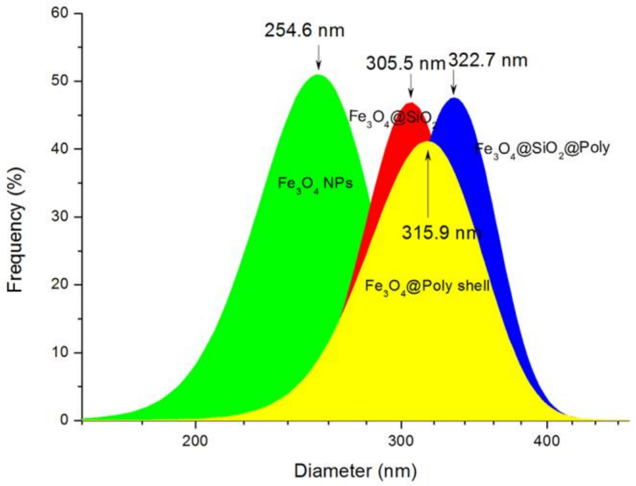

Figure S1. DLS measure of different kinds of nanoparticles.

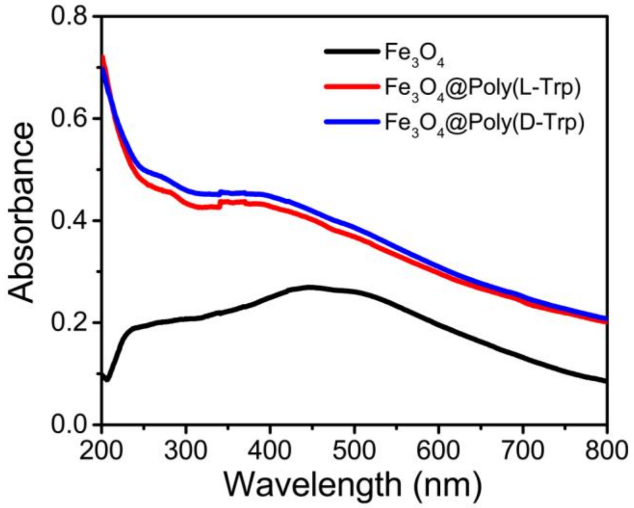

Figure S2. Absorption spectra of Fe_3O_4, Fe_3O_4@Poly(L-Trp) and Fe_3O_4@Poly(D-Trp).

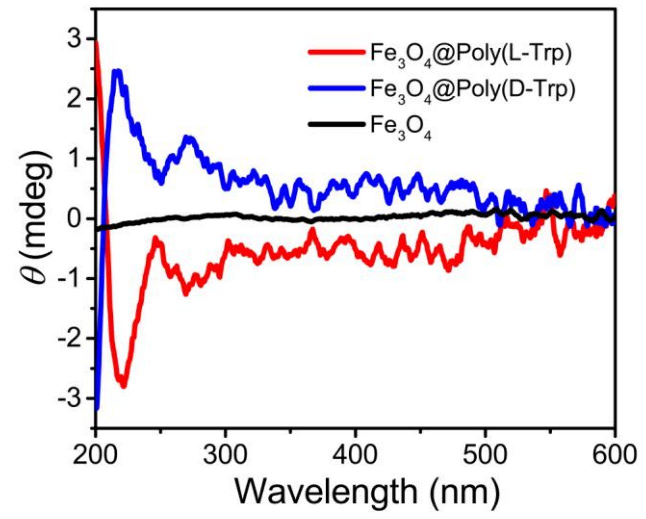

Figure S3. CD spectra of Fe_3O_4, Fe_3O_4@Poly(L-Trp) and Fe_3O_4@Poly(D-Trp).

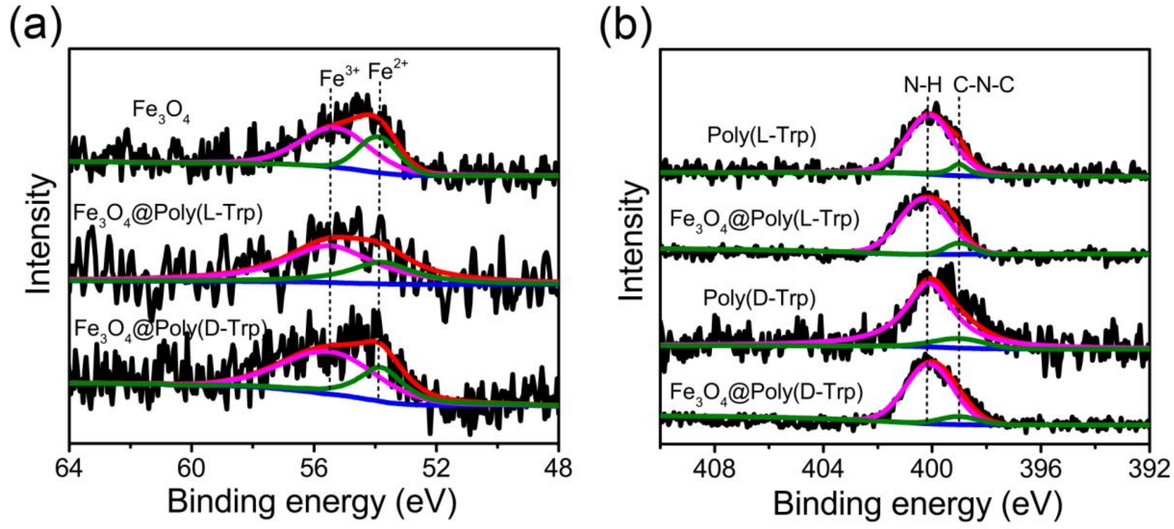

Figure S4. (a) Fe 3p and (b) N 1s XPS spectra.

As decided by Fe 3p spectra, the ratio of Fe^{3+} and Fe^{2+} in final materials was close to 2, which was similar with the bare Fe_3O_4 NPs. N 1s spectra of the polymer shell in final samples also kept unaltered compared with the independent polymer.

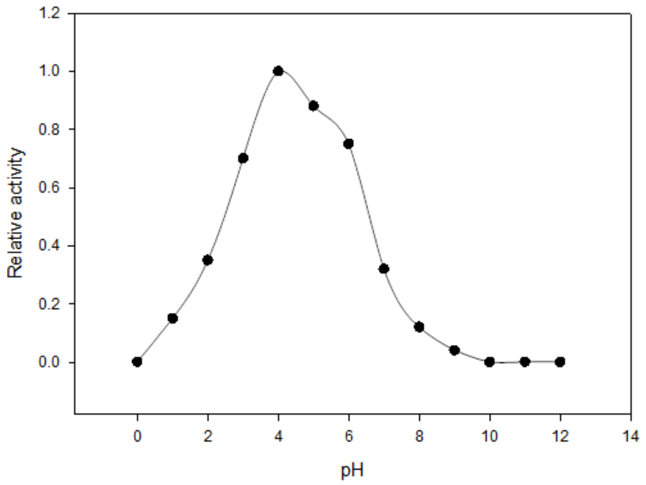

Figure S5. Peroxidase activity of Fe_3O_4@Poly(L-Trp) under different pH values

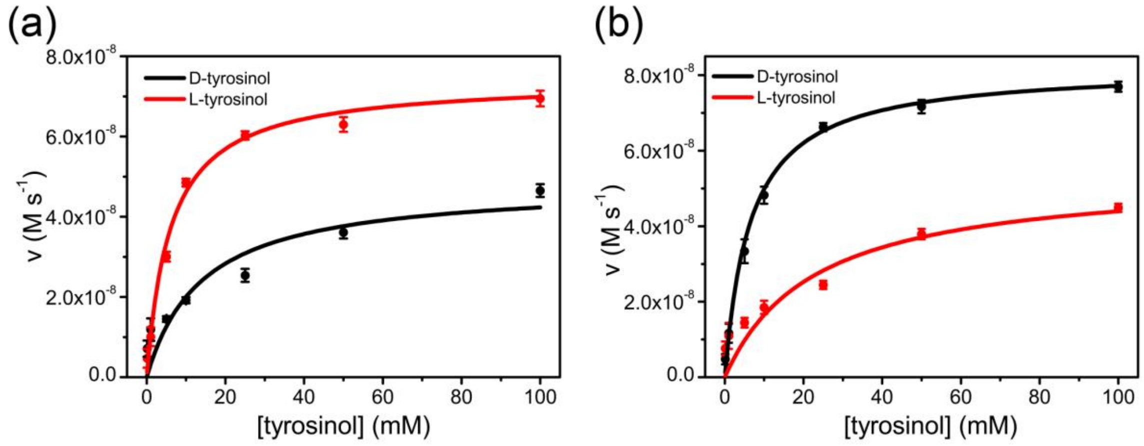

Figure S6. Saturation curves corresponding to the oxidation rate of tyrosinol at variable concentrations D-tyrosinol or L-tyrosinol, in the presence of (a) Fe_3O_4@Poly(L-Trp) and (b) Fe_3O_4@Poly(D-Trp). Details were described in experimental section.

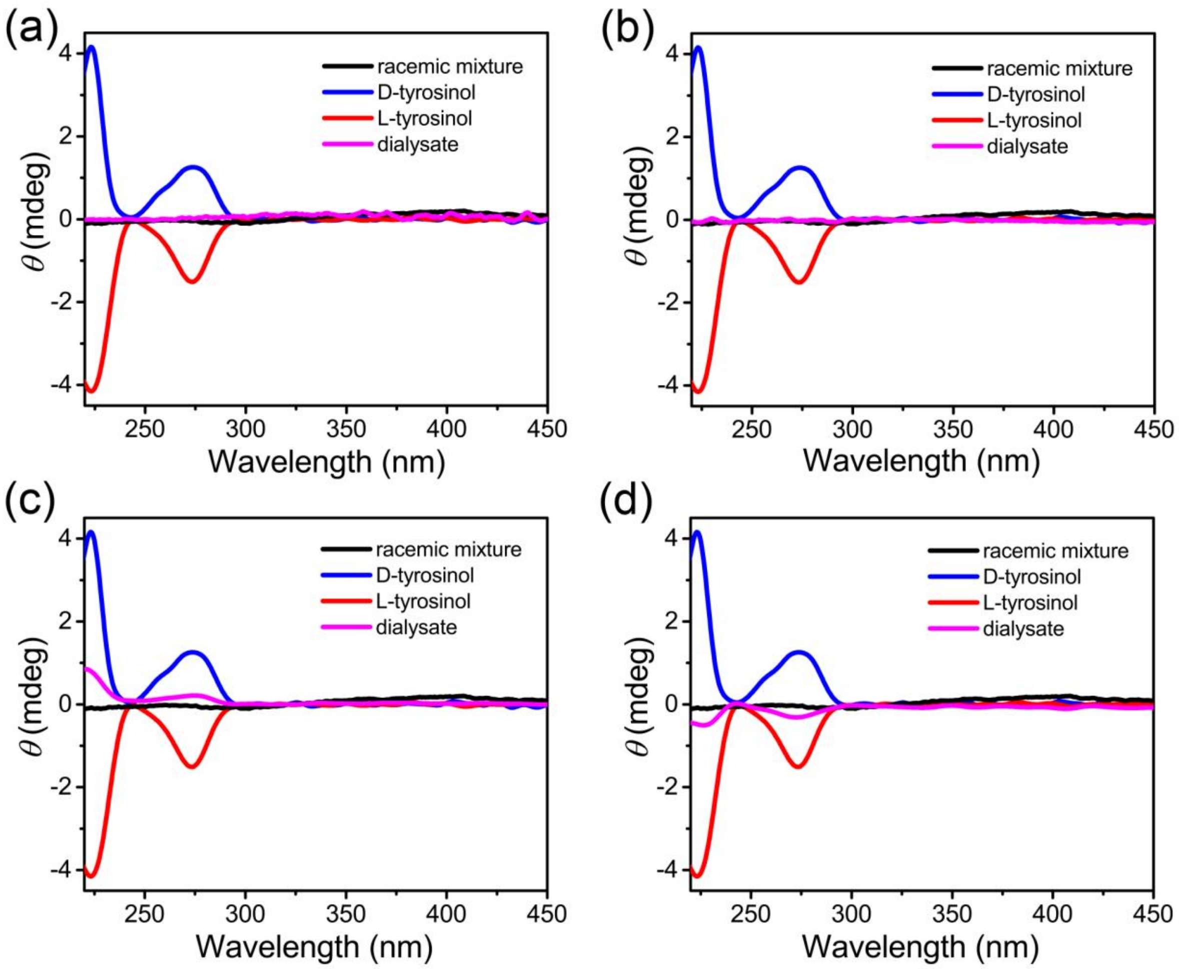

Figure S7. CD spectra of the dialysate for enrichment in the enantiomer with poorer affinity for the a) blank, b) Fe_3O_4, c) Fe_3O_4@Poly(L-Trp) or d) Fe_3O_4@Poly(D-Trp) contained within the dialysis tube in a competition dialysis experiment. 1 ml of different yolk-shell nanozymes (1 mg mL^{-1}) was dialyzed against a 10 ml solution containing a 1:1 mixture of tyrosinol (2.5 mM). After 24 h dialysis, circular dichroism (CD) spectra of the dialysate solutions were measured to determine the enrichment of particular enantiomeric forms.

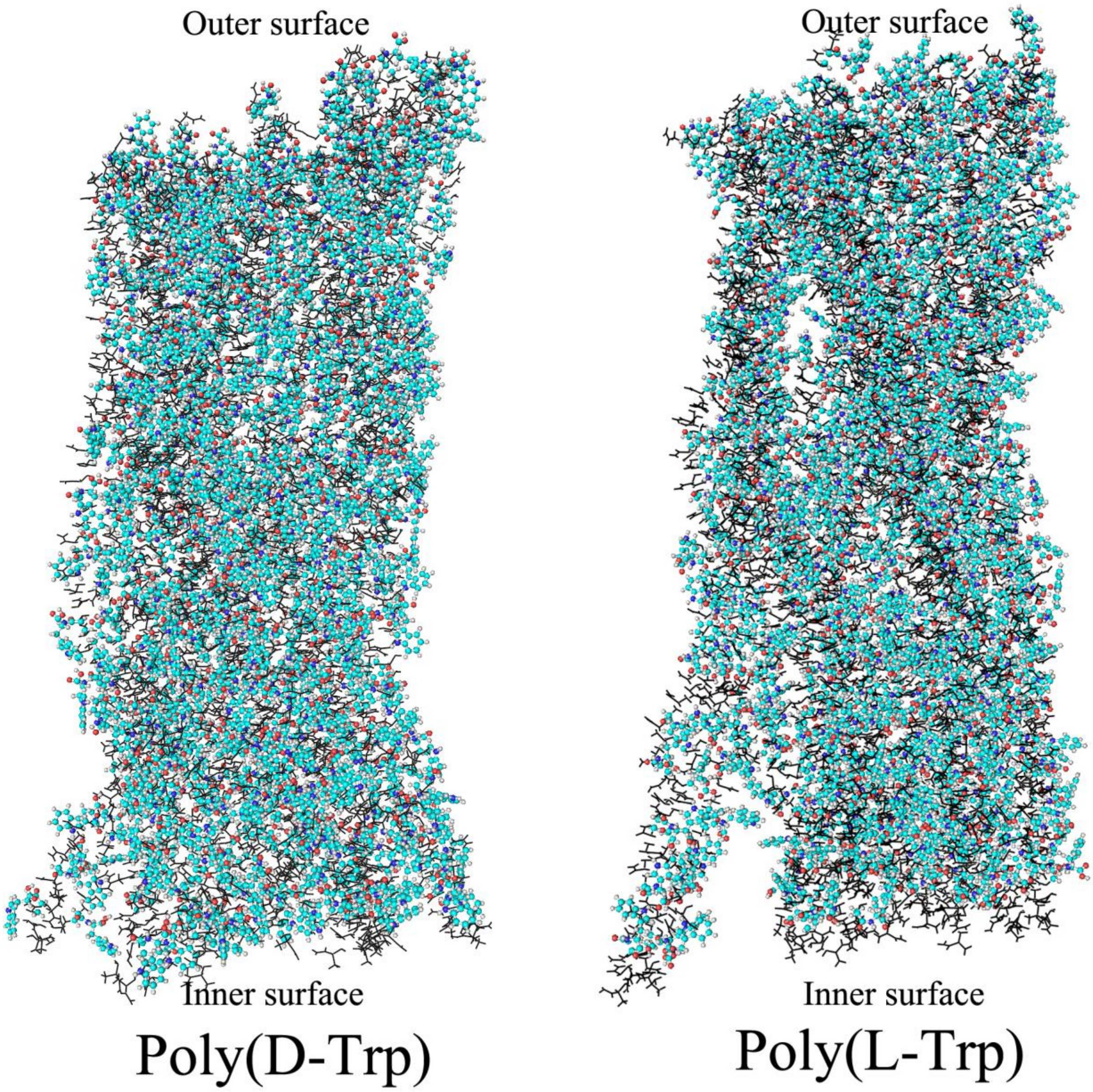

Figure S8. A patch of shell model of Poly(D/L-Trp). Monomers with D/L-Trp amino acid are shown as Ball_and_stick model and all others are shown as black line.

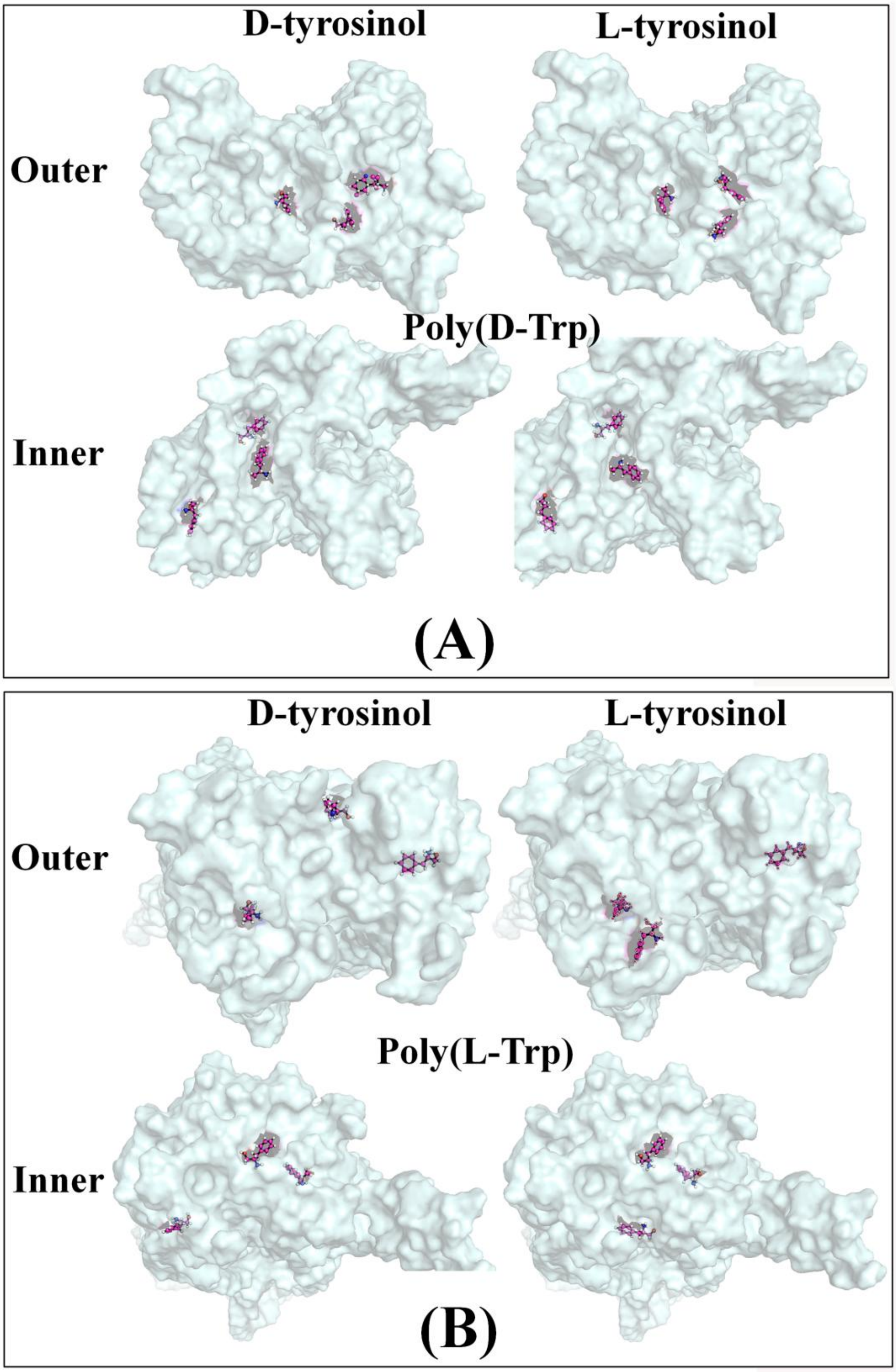

Figure S9. The binding structure models of D/L-tyrosinol located at three different sites of outer and inner surface of (A) Poly(D-Trp) and (B) Poly(L-Trp). The outer and inner surfaces of Poly(D/L-Trp) are shown as palecyan surface and D/L-tyrosinol is shown as ball_and_stick model, carbon as lightmagenta, nitrogen as blue, oxygen as red and hydrogen as white.

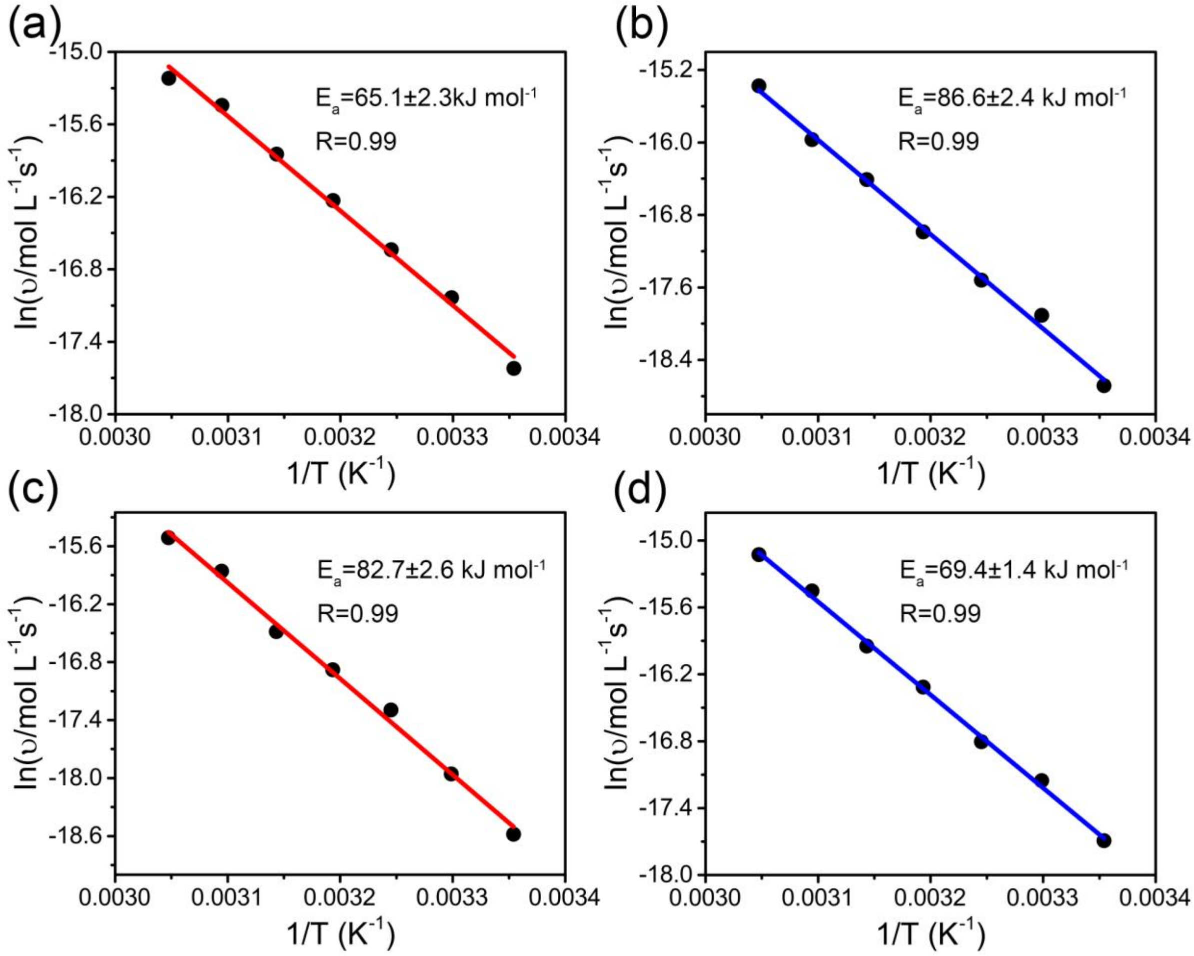

Figure S10. Logarithmic reaction rate of D-tryosinol (red) or L-tyrosinol (blue) oxidation by H_2O_2 with Fe_3O_4@Poly(D-Trp) or Fe_3O_4@Poly(L-Trp) (c, d) as a function of reiprocal temperature. The activation energies (E_a) was calculated according to the Arrhenius equation: $\ln(\nu) = A\text{-}E_a/R\times 1/T$, where A is the frequency factor, R is the gas constant and T is the absolute temperature (K).

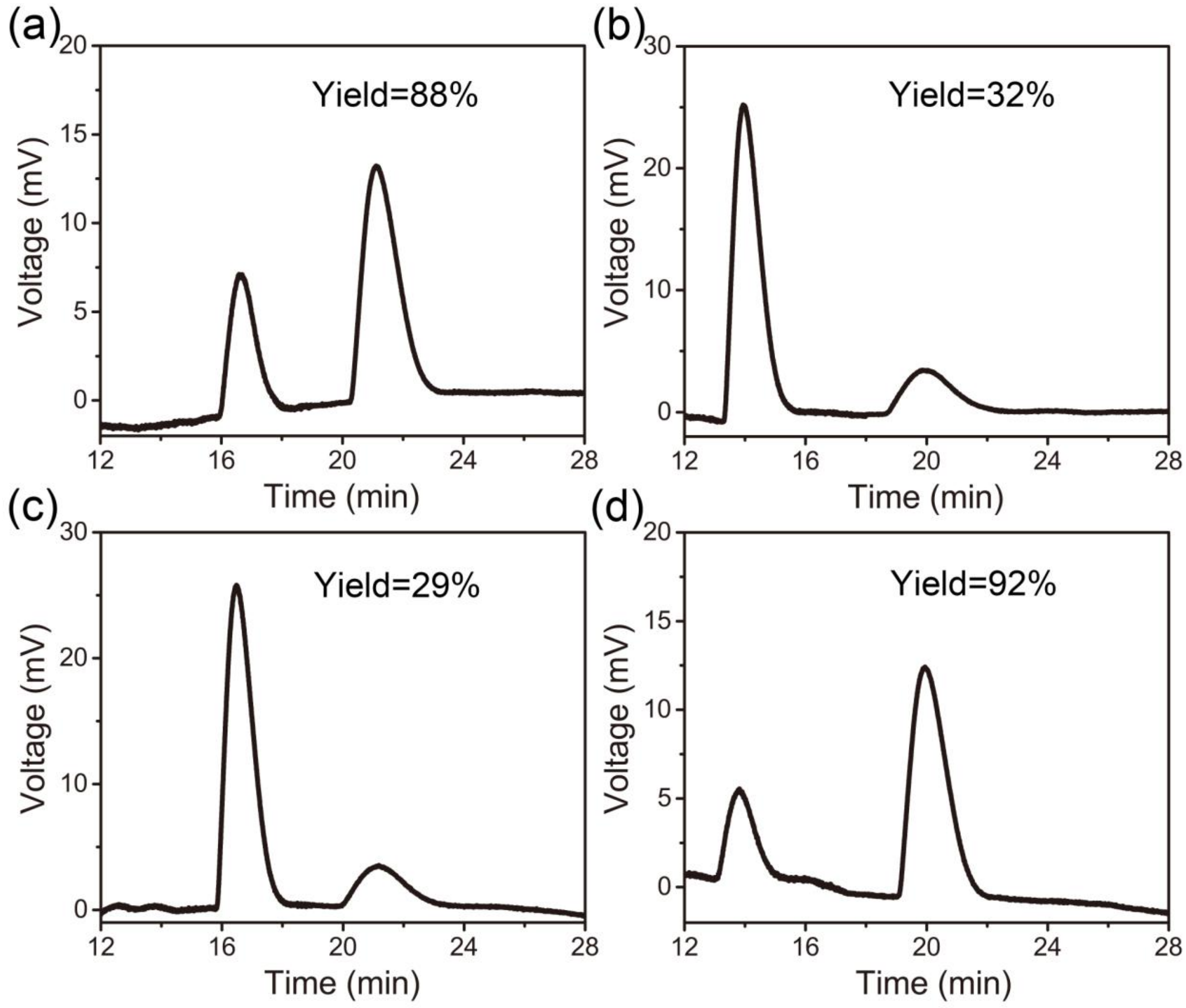

Figure S11. HPLC chromatograms and the corresponding yield of the reaction mixture of L-tyrosinol (a, c) or D-tyrosinol (b, d) catalyzed with Fe_3O_4@Poly(L-Trp) (a, b) and Fe_3O_4@Poly(D-Trp) (c, d).

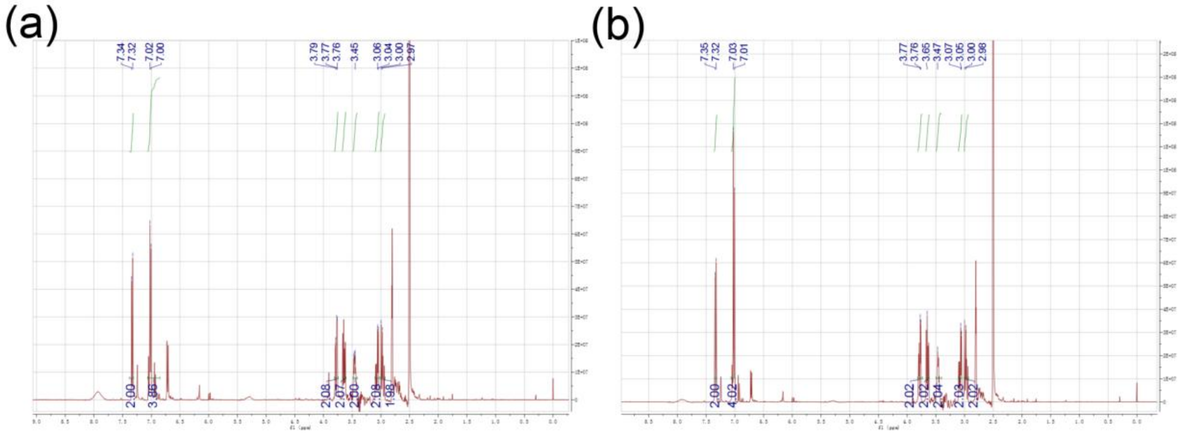

Figure S12. The ^{1}H NMR spectra for the products in the oxidation of (a) L-tyrosinol catalyzed with Fe_3O_4@Poly(L-Trp) or (b) D-tyrosinol catalyzed with Fe_3O_4@Poly(D-Trp).

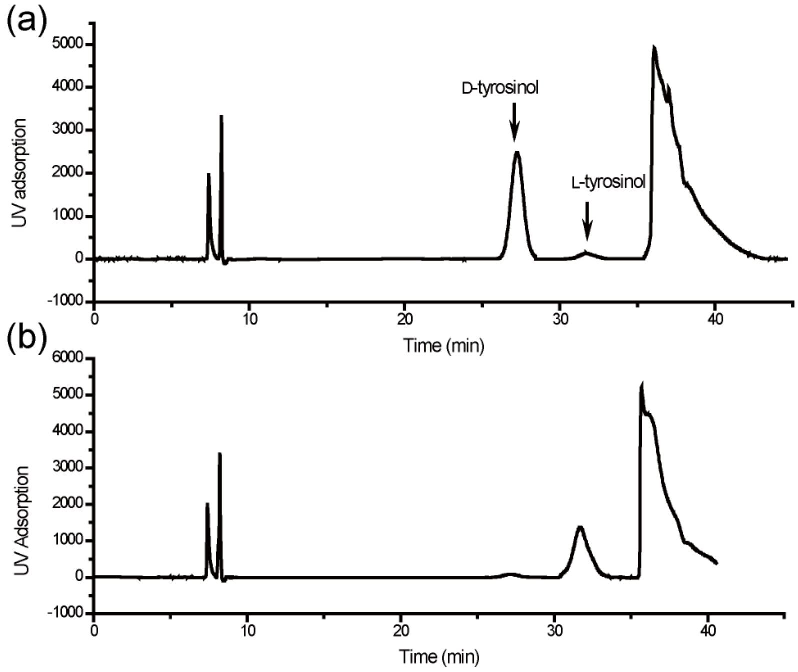

Figure S13. Chiral HPLC chromatograms of the reaction mixture of tyrosinol racemate catalyzed with (a) Fe_3O_4@Poly(L-Trp) and (b) Fe_3O_4@Poly(D-Trp).

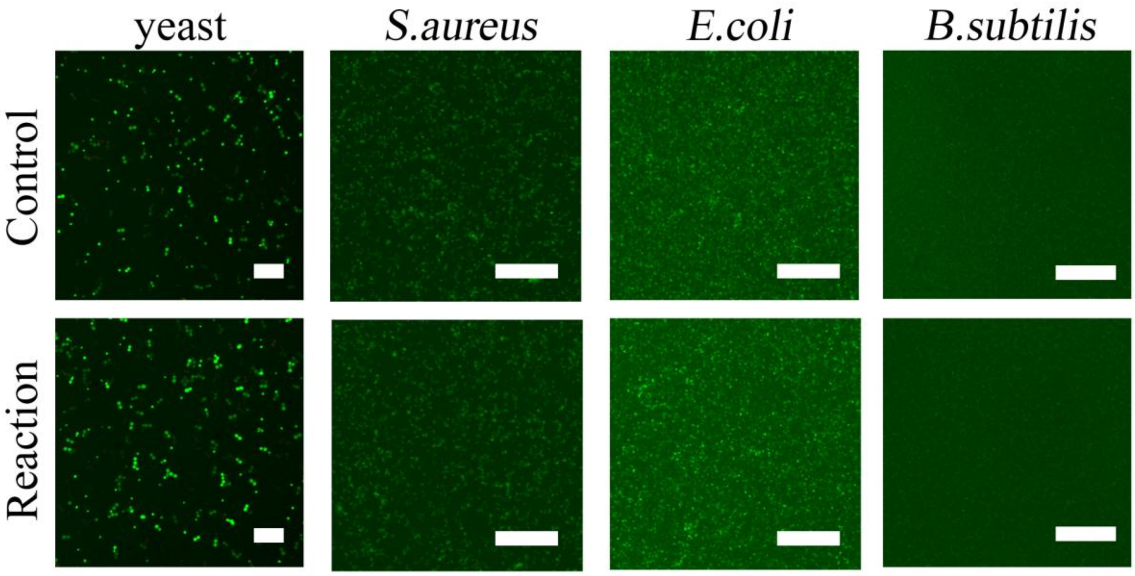

Figure S14. Fluorescence microscopy images of yeast, *S.aureus*, *E.coli* and *B.subtilis* bacteria cells with different conditions. Viable bacteria cells were stained green with calcein, dead bacteria cells were stained red with propidium iodide (PI). (Scale bars: 100 μm.) The reaction bacteria cells were treated with 100 μg mL^{-1} Fe_3O_4@poly(L-/D-Trp), H_2O_2(100 μM) and incubated at 37 ℃ for 30 min.

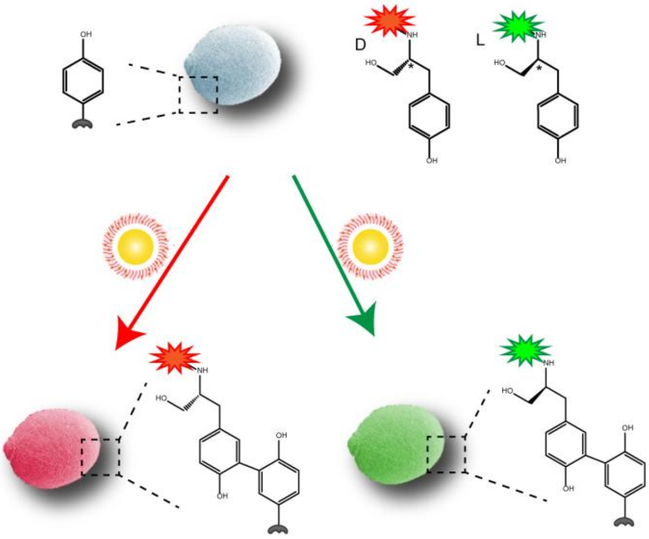

Scheme S1. The surface of yeast cells was labeled with FITC-L-tyrosinol or RhB-D-tyrosinol under the catalysis of our designed artificial enzymes.

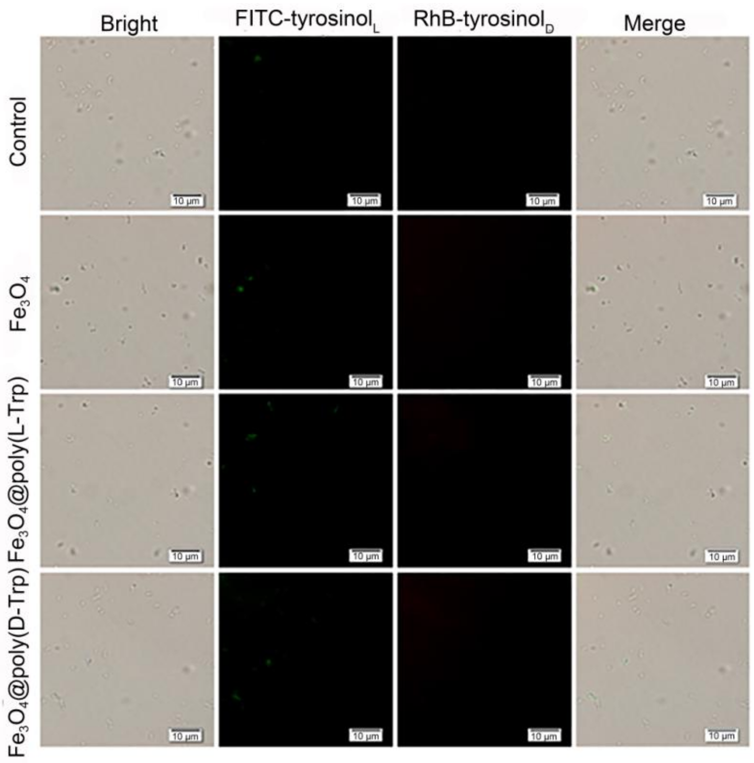

Figure S15. Fluorescence microscopy of *S.aureus* cells treated with H_2O_2, FITC-tyrosinol$_L$ and RhB-tyrosinol$_D$ under no nanozyme (control), Fe_3O_4, Fe_3O_4@poly(L-Trp) or Fe_3O_4@poly(D-Trp) respectively.

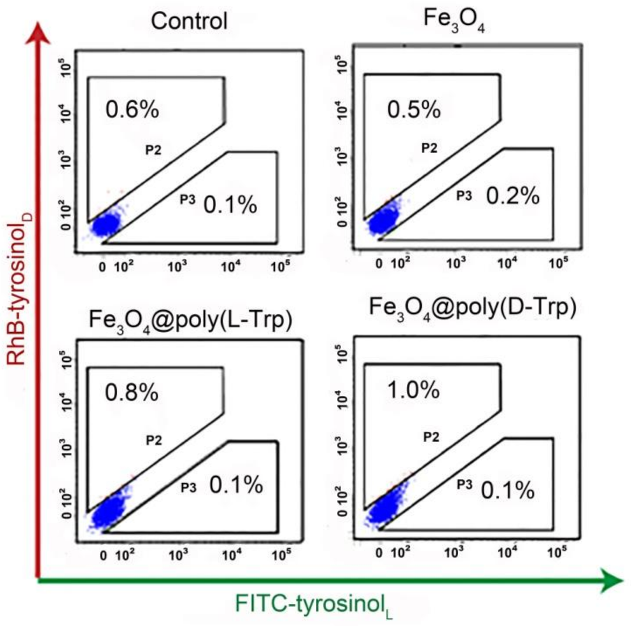

Figure S16. Flow cytometry analysis of *S.aureus* cells treated with H_2O_2, FITC-tyrosinol$_L$ and RhB-tyrosinol$_D$ under no nanozyme (control), Fe_3O_4, Fe_3O_4@poly(L-Trp) or Fe_3O_4@poly(D-Trp) respectively.

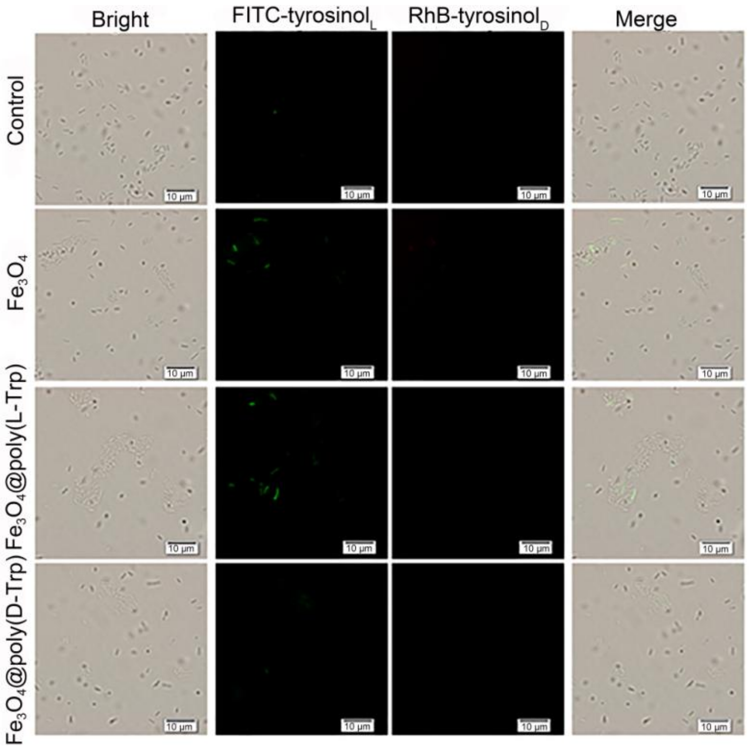

Figure S17. Fluorescence microscopy of *E.coli* cells treated with H_2O_2, FITC-tyrosinol$_L$ and RhB-tyrosinol$_D$ under no nanozyme (control), Fe_3O_4, Fe_3O_4@poly(L-Trp) or Fe_3O_4@poly(D-Trp) respectively.

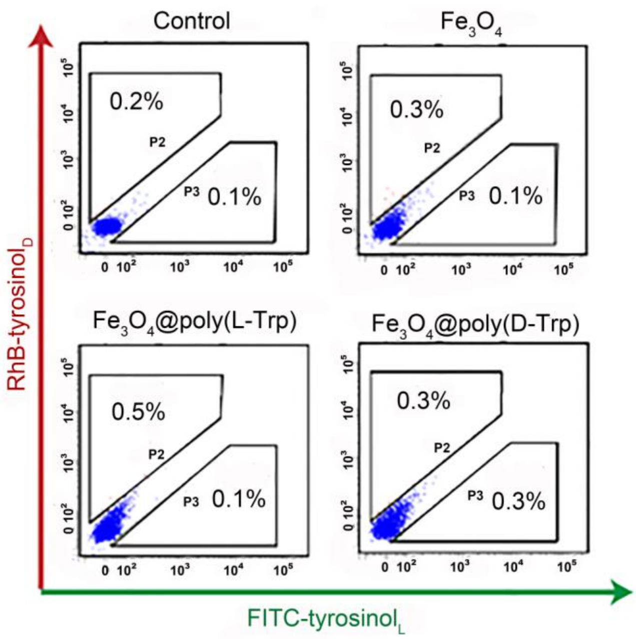

Figure S18. Flow cytometry analysis of *E.coli* cells treated with H_2O_2, FITC-tyrosinol$_L$ and RhB-tyrosinol$_D$ under no nanozyme (control), Fe_3O_4, Fe_3O_4@poly(L-Trp) or Fe_3O_4@poly(D-Trp) respectively.

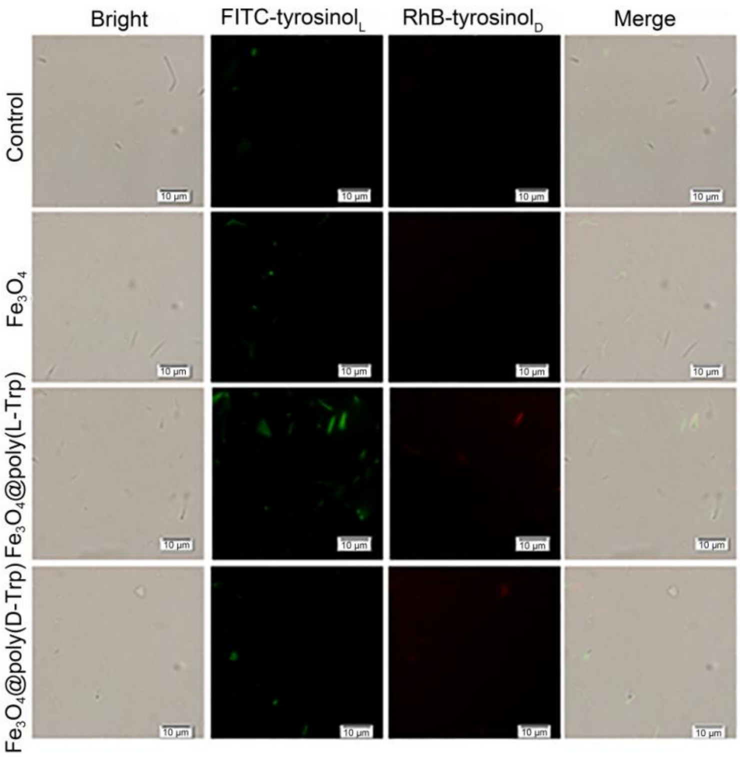

Figure S19 Fluorescence microscopy of *B.subtilis* cells treated with H_2O_2, FITC-tyrosinol$_L$ and RhB-tyrosinol$_D$ under no nanozyme (control), Fe_3O_4, Fe_3O_4@poly(L-Trp) or Fe_3O_4@poly(D-Trp) respectively.

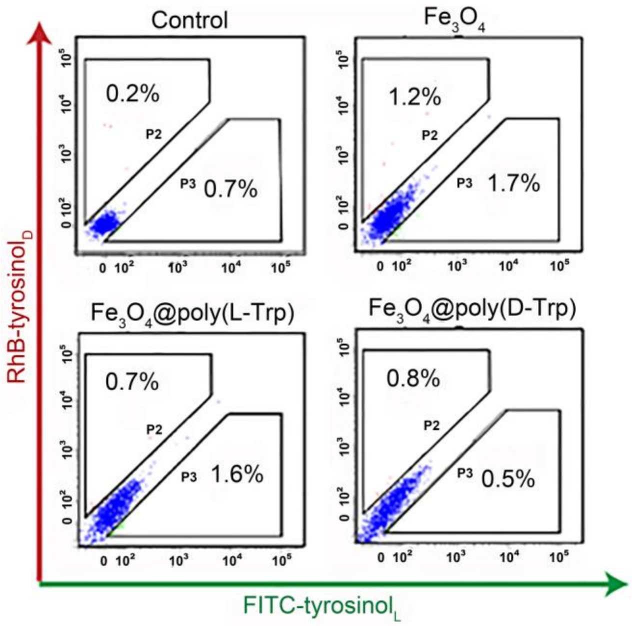

Figure S20. Flow cytometry analysis of *B.subtilis* cells treated with H_2O_2, FITC-tyrosinol$_L$ and RhB-tyrosinol$_D$ under no nanozyme (control), Fe_3O_4, Fe_3O_4@poly(L-Trp) or Fe_3O_4@poly(D-Trp) respectively.

References:

1 (a) E. Golub, H. B. Albada, W.-C. Liao, Y. Biniuri and I. Willner, *J. Am. Chem. Soc.* 2016, **138**, 164; (b) Y. Sun, C. Zhao, N. Gao, J. Ren and X. Qu, *Chem.-Eur. J.* 2017, **23**, 18146; (c) H. Sun, A. Zhao, N. Gao, K. Li, J. Ren and X. Qu, *Angew. Chem.-Int. Edit.* 2015, **54**, 7176.

2 E. Antipov, A. E. Cho, K. D. Wittrup and A. M. Klibanov, *P. Natl. Acad. Sci. USA* 2008, **105**, 17694.

3 C. Xu, C. Zhao, M. Li, L. Wu, J. Ren and X. Qu, *Small* 2014, **10**, 1841.

4 P. Zhan, Z.-G. Wang, N. Li and B. Ding, *ACS Catal.* 2015, **5**, 1489.

5 J. L. Chen, C. Pezzato, P. Scrimin and L. J. Prins, *Chem.-Eur. J.* 2016, **22**, 7028.

6 Y. Zhou, H. Sun, H. Xu, S. Matysiak, J. Ren and X. Qu, *Angew. Chem. Int. Ed.* 2018, **57**, 16791-16795.

7 M. J. Frisch, G. W. Trucks, H. B. Schlegel, G. E. Scuseria, M. A. Robb, J. R. Cheeseman, G. Scalmani, V. Barone, B. Mennucci, G. A. Petersson, et. al. Gaussian 09; Gaussian, Inc.: Wallingford, CT, USA, 2009.

8 J. Wang, R. M. Wolf, J. W. Caldwell, P. A. Kollman and D. A. *J.* Case, *J. Comput. Chem.* 2004, **25**, 1157.

9 F.-Y. Dupradeau, A. Pigache, T. Zaffran, C. Savineau, R. Lelong, N. Grivel, D. Lelong, W. Rosanski and P. Cieplak, *Phys. Chem. Chem. Phys.* 2010, **12**, 7821.

10 O. Trott and A. J. *J.* Olson, *J. Comput. Chem.* 2010, **31**, 455.

11 (a) J. Baxter, *J. Oper. Res. Soc.* 1981, **32**, 815; (b) C. Blum, A. Roli and M. Sampels, Hybrid metaheuristics: An emerging approach to optimization Springer-Verlag, Berlin, Heidelberg, 2008.

12 J. Srinivasan, T. E. Cheatham, P. Cieplak, P. A. Kollman and D. A. Case, *J. Am. Chem. Soc.* 1998, **120**, 9401.

13 D. A. Case, T. A. Darden, I. T. E. Cheatham, C. L. Simmerling, J. Wang, R. E.Duke, R. Luo, R. C. Walker, W. Zhang, K. M. Merz, et. al. Amber12. 2012.

14 B. R. Miller, T. D. McGee, J. M. Swails, N. Homeyer, H. Gohlke and A. E. Roitberg, *J. Chem. Theory Comput.* 2012, **8**, 3314.

15 (a) T. Hou, Y. Li and W. Wang, *Bioinformatics* 2011, **27**, 1814; (b) T. Hou and R. Yu, *J. Med. Chem.* 2007, **50**, 1177.

16 (a) W. G. Hoover, *Phys. Rev. A* 1985, **31**, 1695; (b) S. Nosé and M. L. Klein, *Mol. Phys.* 1983, **50**, 1055; (c) G. J. Martyna, D. J. Tobias and M. L. Klein, *J. Chem. Phys.* 1994, **101**, 4177; (d) S. E. Feller, Y. Zhang, R. W. Pastor and B. R. Brooks, *J. Chem. Phys.* 1995, **103**, 4613.

17 (a) P. P. Ewald, *Annalen der Physik* 1921, **369**, 253; (b) T. Darden, D. York and L. Pedersen, *J. Chem. Phys.* 1993, **98**, 10089.

18 (a) J. P. Ryckaert, G. Ciccotti and H. J. C. Berendsen, *J. Comput. Phys.* 1977, **23**, 327; (b) Y. Weinbach and R. Elber, *J. Comput. Phys.* 2005, **209**, 193.

19 J. C. Phillips, R. Braun, W. Wang, J. Gumbart, E. Tajkhorshid, E. Villa, C. Chipot, R. D. Skeel, L. Kalé and K. Schulten, *J. Comput. Chem.* 2005, **26**, 1781.

NMR spectra:

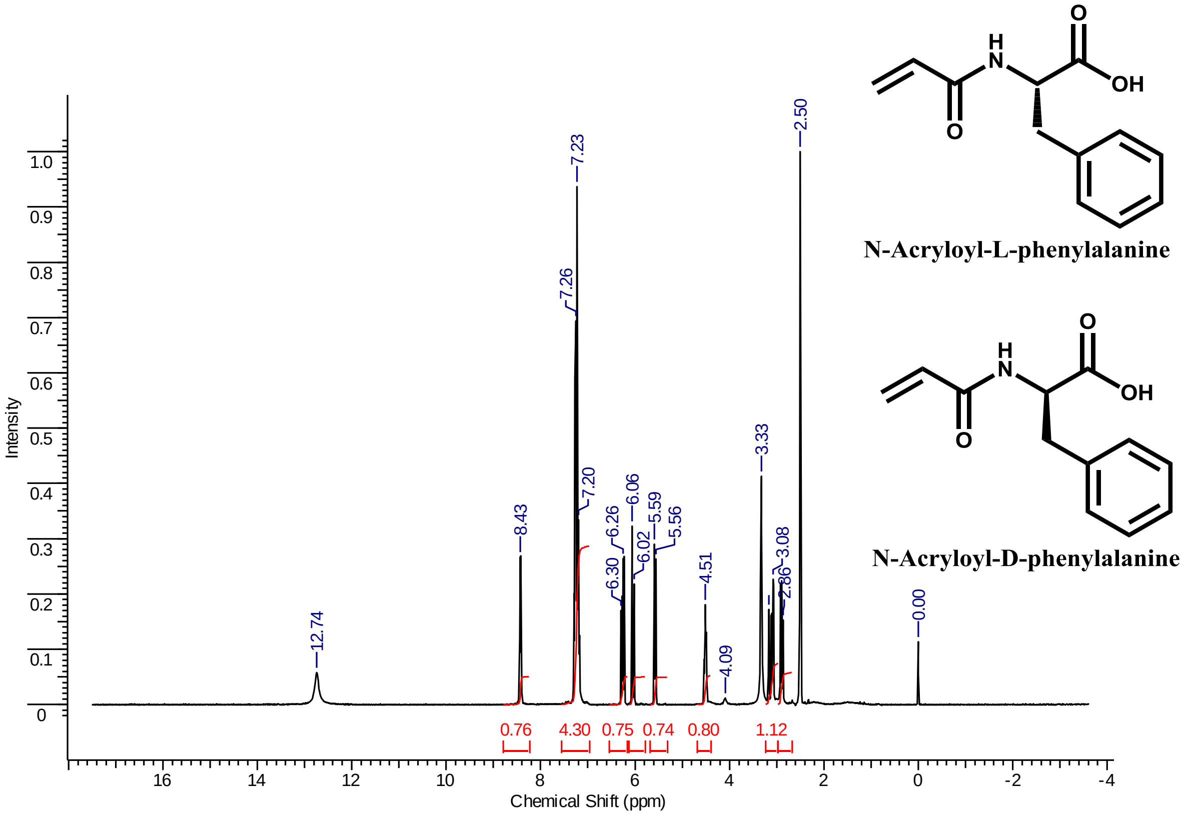
12.74
8.43
7.26
7.23
7.20
6.30
6.26
6.06
6.02
5.59
5.56
4.51
4.09
3.33
3.08
2.86
2.50
0.00
0.76
4.30
0.75
0.74
0.80
1.12
Intensity
Chemical Shift (ppm)
O
H
N
OH
O
N-Acryloyl-L-phenylalanine
N-Acryloyl-D-phenylalanine

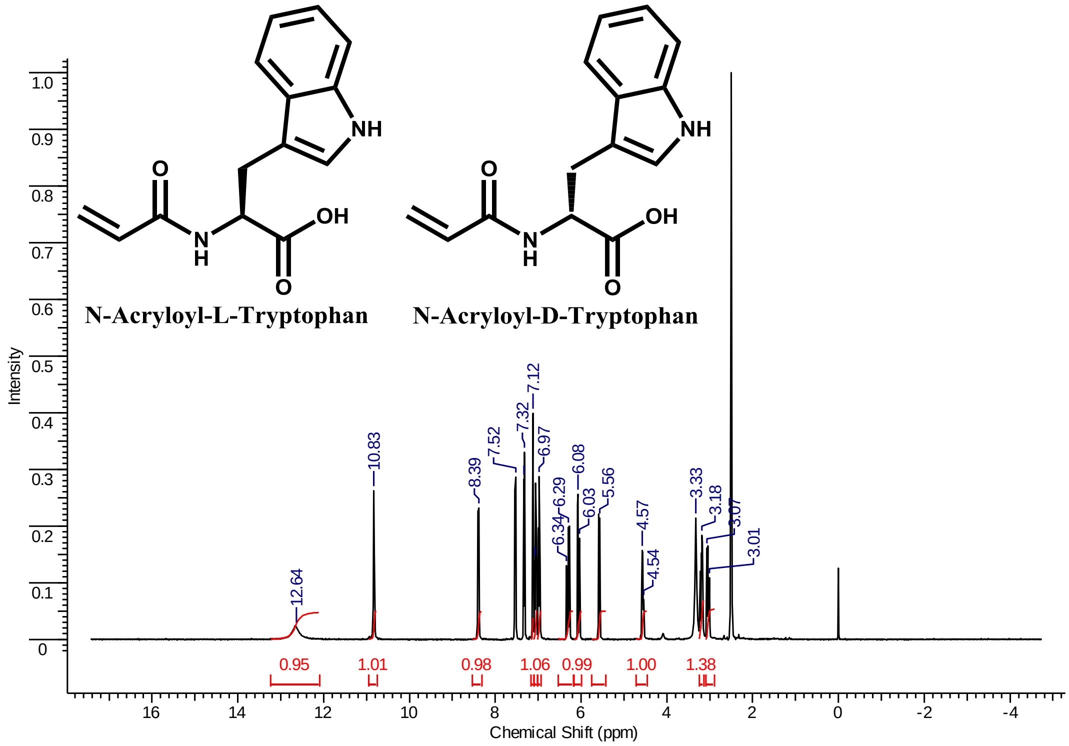
N-Acryloyl-L-Tryptophan
N-Acryloyl-D-Tryptophan
Intensity
Chemical Shift (ppm)
12.64
10.83
8.39
7.52
7.32
7.12
6.97
6.34
6.29
6.08
6.03
5.56
4.57
4.54
3.33
3.18
3.07
3.01
0.95
1.01
0.98
1.06
0.99
1.00
1.38

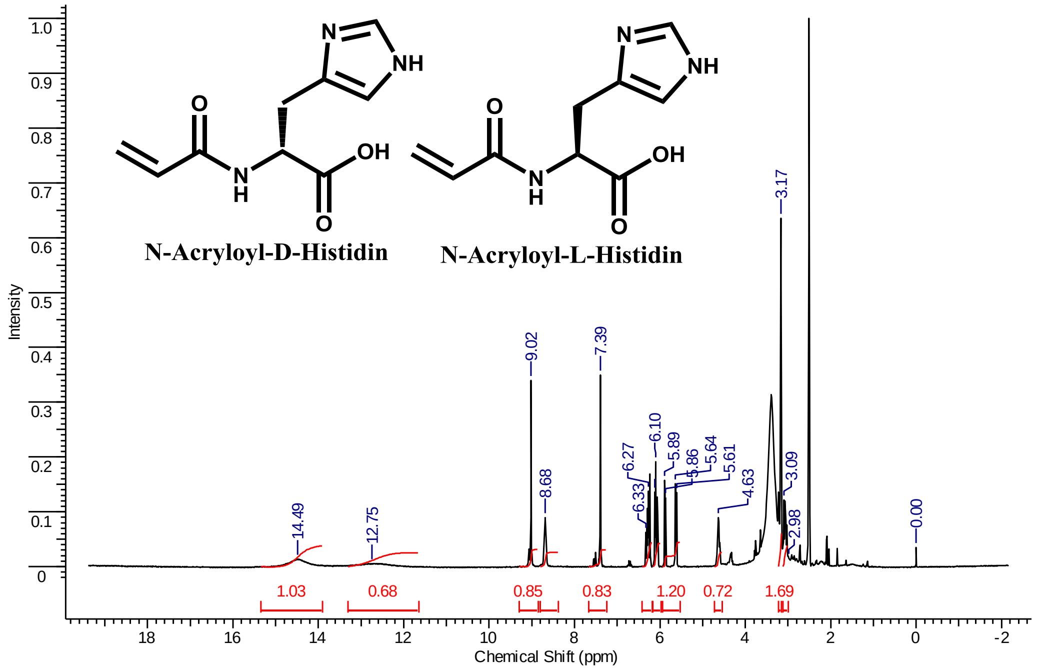

N-Acryloyl-D-Histidin
N-Acryloyl-L-Histidin
Intensity
Chemical Shift (ppm)
14.49
12.75
9.02
8.68
7.39
6.33
6.27
6.10
5.89
5.86
5.64
5.61
4.63
3.17
3.09
2.98
0.00
1.03
0.68
0.85
0.83
1.20
0.72
1.69

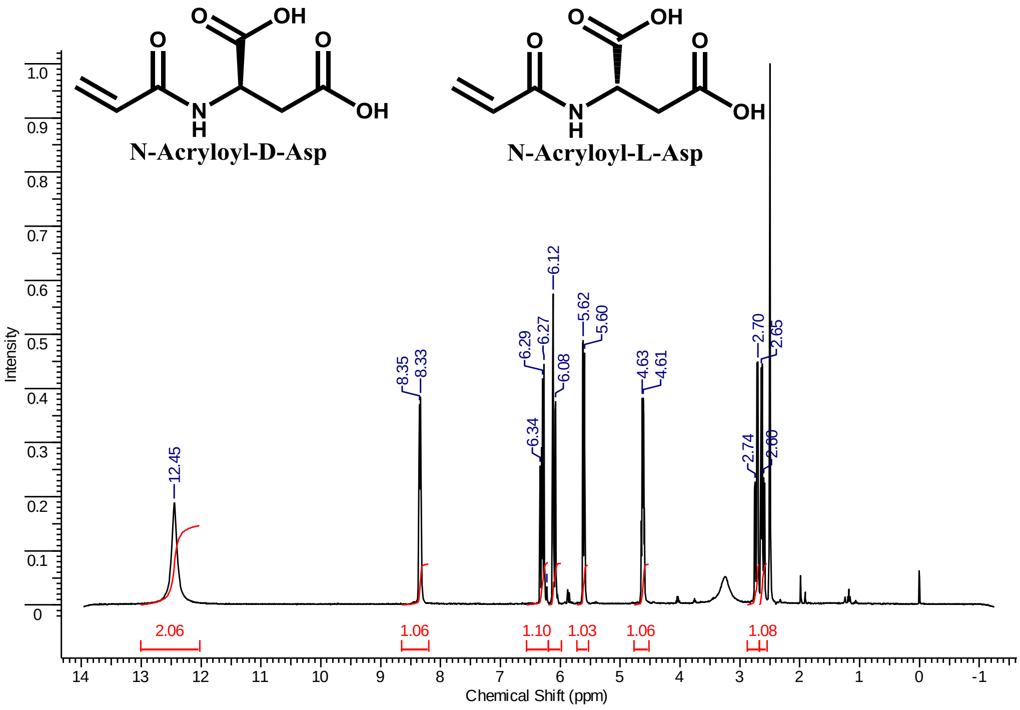

N-Acryloyl-D-Asp
N-Acryloyl-L-Asp
Intensity
Chemical Shift (ppm)
12.45
8.35
8.33
6.34
6.29
6.27
6.12
6.08
5.62
5.60
4.63
4.61
2.74
2.70
2.65
2.60
2.06
1.06
1.10
1.03
1.06
1.08
